# Identifying determinants of varenicline adherence using the Theoretical Domains framework: a rapid review

**DOI:** 10.1186/s12889-024-18139-z

**Published:** 2024-03-04

**Authors:** Nadia Minian, Melissa Wong, Sowsan Hafuth, Terri Rodak, Alma Rahimi, Dea Gjomema, Jonathan Rose, Laurie Zawertailo, Matt Ratto, Peter Selby

**Affiliations:** 1 INTREPID Lab (Formerly Nicotine Dependence Service), Centre for Addiction and Mental Health, 1025 Queen St W, Toronto, ON M6H 1H4 Canada; 2 Department of Family and Community Medicine, University of Toronto, Toronto, ON Canada; 3 Campbell Family Mental Health Research Institute, Centre for Addiction and Mental Health, Toronto, ON Canada; 4 Department of Pharmacology and Toxicology, University of Toronto, Toronto, ON Canada; 5 Institute of Medical Sciences, University of Toronto, Toronto, ON Canada; 6 Department of Education, CAMH Library, Centre for Addiction and Mental Health, Toronto, ON Canada; 7 Department of Electrical and Computer Engineering, The Edward S. Rogers Sr, University of Toronto, Toronto, ON Canada; 8 Faculty of Information Bell University Labs Chair in Human-Computer Interaction Faculty Affiliate, Schwartz-Reisman Institute for Technology and Society, University of Toronto, Toronto, ON Canada; 9 Department of Psychiatry, University of Toronto, Toronto, ON Canada; 10 Dalla Lana School of Public Health, University of Toronto, Toronto, ON Canada

**Keywords:** Varenicline, Medication Adherence, Smoking Cessation, Theoretical Domains Framework, Behaviour Change Techniques, Barriers, Facilitators

## Abstract

**Background:**

Adhering to varenicline has been shown to significantly improve the chances of successfully quitting smoking, with studies indicating a twofold increase in 6-month quit rates. However, despite its potential benefits, many individuals struggle with maintaining good adherence to varenicline; thus there is a need to develop scalable strategies to help people adhere. As a first step to inform the development of an intervention to improve adherence to varenicline, we conducted a rapid literature review to identify: 1) modifiable barriers and facilitators to varenicline adherence, and 2) behaviour change techniques associated with increased adherence to varenicline.

**Methods:**

We searched MEDLINE, Embase, APA PsycINFO, CINAHL, and the Cochrane Central Register of Controlled Trials for relevant studies published between 2006 and 2022. Search terms included “varenicline,” “smoking cessation,” and "adherence," and their respective subject headings and synonyms. We screened and included studies reporting modifiable determinants of adherence to varenicline and then assessed quality, extracted modifiable determinants and mapped them to the Theoretical Domains Framework version 2 and the Behaviour Change Technique Taxonomy version 1.

**Results:**

A total of 1,221 titles were identified through the database searches; 61 met the eligibility criteria. Most of the studies were randomized controlled trials and predominantly focused on barriers to varenicline. Only nine studies explicitly mentioned behaviour change techniques used to help varenicline adherence. Eight domains were identified as barriers to varenicline adherence (behavioural regulation, memory, goals, intentions, beliefs about capabilities, beliefs about consequences, optimism/pessimism, and environmental context) and five as facilitators (knowledge, behavioural regulation, beliefs about capabilities, social influences, and environmental context).

**Conclusions:**

This study identifies barriers and facilitators that should be addressed when developing a complex adherence intervention tailored to patients’ needs based on modifiable determinants of medication adherence, some of which are under- used by existing adherence interventions. The findings from this review will inform the design of a theory-based healthbot planned to improve varenicline adherence in people undergoing smoking cessation treatment.

**Systematic review registration:**

This study was registered with PROSPERO (# CRD42022321838).

**Supplementary Information:**

The online version contains supplementary material available at 10.1186/s12889-024-18139-z.

## Background

Tobacco use and exposure results in more than eight million deaths worldwide each year [1], prompting an urgent need to implement interventions to promote smoking cessation. There are currently three pharmacotherapies approved for smoking cessation by the US Food and Drug Administration (FDA): varenicline, bupropion, and nicotine replacement therapy (NRT) [2]. A Cochrane systematic review reported that, compared to bupropion or NRT, varenicline is the most effective pharmacotherapy for maintaining long-term smoking abstinence (at six months or more) [3]. A high-affinity partial agonist at the α4β2 nicotinic acetylcholine receptor, varenicline decreases the rewarding effects of tobacco through its dual effects as an agonist by binding to the receptor to reduce craving and as an antagonist by competing with nicotine for the receptor [2, 4]. Despite varenicline being superior to other pharmacotherapy in the treatment of tobacco dependence, low adherence to varenicline is a significant obstacle to the success of this smoking cessation treatment [5]. Meta-analyses have demonstrated the association between varenicline and adverse effects such as nausea, constipation, flatulence [6], sleeping disorders, insomnia, abnormal dreams, and fatigue [7]. In a retrospective cohort study examining varenicline adherence, 55% of the study participants never began their 12-week treatment, 20% began but failed to complete their treatment, and only 25% of the participants adhered to and completed their treatment [5].

Studies have shown that providing behavioural supports and tailored interventions can increase adherence to smoking cessation medications [8].These studies have a large variability in the strengths of effects [9–12] which may be accounted for by the active ingredients in the behavioural supports the intervention offered. In addition, there is no review examining the behaviour change theory that could guide the design of the intervention targeting varenicline adherence. This is a significant shortcoming given that there is growing evidence, including the UK Medical Research Council's (MRC) framework for complex interventions [13] supporting the use of theory in complex interventions. Theory holds the potential to enhance researchers' comprehension of the behavior change process and provide guidance in the development and refinement of interventions [14]. For instance, theory can help identify theoretical constructs to target within the intervention (e.g. 'optimism.'). Therefore, before designing an intervention to help people adhere to their varenicline treatment, it is essential to conduct a review exploring modifiable determinants that influence varenicline adherence, grounded in a theoretical framework. The Behaviour Change Technique Taxonomy version 1 (BCTTv1) provides a practical taxonomy to describe the active content of an intervention [15]. Behaviour change techniques (BCTs) can be mapped to the Theoretical Domains Framework (TDF) [16], a framework that integrates 33 theories and 128 constructs into a single framework that contains 14 domains [17]. The TDF, in turn, can be mapped to a well-established model of behaviour change: the Capability, Opportunity, and Motivation Model of Behaviour (COM-B). COM-B suggests that behaviour change results from an interaction between people’s capability, motivation, and opportunities for the behaviour [18].

The aim of this rapid review is twofold: 1) to identify the modifiable barriers and facilitators to varenicline adherence in people using varenicline for smoking cessation, and 2) to identify the behaviour change techniques associated with helping people adhere to their varenicline treatment.

The findings from this review will inform the design of a theory-based healthbot planned to improve varenicline adherence in people undergoing smoking cessation treatment.

## Methods

We chose to conduct a rapid review since it is a timely, cost-effective and efficient way to gather high-quality evidence to inform health program decisions [19]. The rapid review was conducted in accordance with the Cochrane rapid review methods recommendations [20] and it is reported following the Preferred Reporting Items for Systematic Reviews and Meta-Analyses (PRISMA) statement (see Additional file 1) [21]. The study was registered with PROSPERO (# CRD42022321838).

### Eligibility criteria

The research question was developed using the PICO model.

- Population: The population of interest were individuals using varenicline for smoking cessation.
- Intervention: Studies were included if varenicline was used as an intervention for smoking cessation. We included studies using multiple smoking cessation medications, as long as they reported factors associated with only varenicline users separately.
- Comparator: In studies with a comparator group, the comparator was either a placebo, an active control group, or no intervention
- Outcome: The outcome of interest was reported modifiable factors associated with adherence to varenicline.

### Exclusion criteria

1. Publications such as commentaries, abstracts, conference papers, reviews, editorial letters, protocols, book chapters, thesis/dissertations, case reports, and case series.
2. Studies that did not separately report barriers and/or facilitators associated directly with varenicline adherence.
3. Studies in which varenicline was not administered for smoking cessation.
4. Non-English language articles.
5. Non-peer reviewed articles.

### Information sources and search strategy

The search strategy was developed with a health sciences librarian (TR), who conducted all searches. The strategy was tested and finalized in MEDLINE (Ovid), then translated and run in the following bibliographic databases: MEDLINE, Embase, Cumulative Index to Nursing & Allied Health Literature (CINAHL), APA PsycInfo, and Cochrane Central Register of Controlled Trials (CENTRAL).

The search strategy was designed to identify the overlap between three concepts: tobacco smoking, varenicline, and treatment adherence (see Additional file 1). The smoking concept was kept broad (e.g. “smoking”, “nicotine”, “tobacco” and relevant subject headings) and functioned only to omit alternative uses of varenicline (i.e. treatment of dry eye syndrome) [22]. The varenicline concept included generic, and brand names (“varenicline”, “Chantix”, “Champix”) searched in the major record fields.

The treatment adherence concept used database-specific subject headings, natural language keywords, and advanced search operators such as truncation and adjacency operators to balance specificity and sensitivity. Variations of search terms such as “retention”, “dropout”, and “compliance” were searched in the title, subject heading, and keyword fields and were linked with “therapy” or “treatment” or “program” using an adjacency operator to search the abstract field. Terms such as “barrier” and “facilitator” were searched in the title, subject heading, and keyword fields and were linked with treatment or retention terms using an adjacency operator to search the abstract field.

The terms and concepts were combined using Boolean operators. Non-human animal studies were excluded [23], as were the following publication types when possible: book chapters, dissertations, conference abstracts, editorials, and letters. Year limit applied was 2006 to the date of the search (May 6, 2022) to reflect the FDA’s approval year of varenicline [24]. The core MEDLINE search strategy can be found in Additional file 2.

The studies located by the research librarian were imported into the reference manager, EndNote [25], and then uploaded into the systematic review software, Covidence [26]. Articles with duplicates were tagged and removed in Covidence [26].

### Study selection process

All reviewers conducted a pilot exercise on Covidence to calibrate and evaluate the review forms used in the title and abstract screening, full-text screening, data extraction, and quality assessment. For the pilot screening, all reviewers conducted title and abstract screening on 39 studies and conducted full-text screening on five randomly selected studies that were included in the title and abstract screening stage [20].

Two reviewers independently screened 201 studies for the title and abstract screening, which included resolved conflicts. Afterwards, one reviewer screened the remaining abstracts while a second reviewer screened the abstracts deemed irrelevant by the first reviewer. Given that modifiable factors associated with varenicline adherence could not always be determined in the title and abstract, only the “yes” and “no” options on Covidence were used in the title and abstract screening, where “yes” was selected if the abstract was ambiguous or suggested the reporting of barriers and/or facilitators to varenicline adherence. Studies with missing abstracts also received a vote for “yes” and eligibility was determined in full-text screening. Two reviewers were required for full-text screening, where one reviewer screened all the included full-text articles (MW) while a second reviewer screened the full-text articles excluded by the first reviewer. Conflicts were resolved by a third reviewer or by consensus [20].

### Data extraction

Utilizing the revised data extraction form from the pilot (see Additional file 3), data extraction was performed by two reviewers. One reviewer extracted data using the data extraction form and a second reviewer verified the accuracy and completeness of the data extracted by the first reviewer. Conflicts were resolved by a third reviewer or by consensus [20]. Missing data were obtained by contacting the corresponding authors of the included studies. Extracted data included:

- 1. Barriers and facilitators associated with varenicline adherence.
- 2. “Active ingredients” employed by the varenicline adherence intervention, defined as the components of a behaviour intervention that are needed for it to work and are observable, replicable and irreducible [25],
- 3. Study information including: sample size, location of intervention, study design, theories used to design the intervention, delivery of intervention, method of smoking cessation (e.g., abrupt cessation, gradual cessation via reduction), and type of tobacco product used.
- 4. Demographic information: gender proportion, target population of intervention, age, race, and any additional demographic information reported.
- 5. Information regarding varenicline adherence including: definition of varenicline adherence, adherence outcome measures (e.g., self-report, pill count), and degree of non-adherence (e.g. discontinuation, reduction). For studies in which adherence to varenicline was not the primary outcome, adherence was defined as adherence to the varenicline treatment. Participants who failed to adhere to their varenicline treatment (e.g., discontinued or stopped taking varenicline but were still in the study) were considered non-adherent.

Barriers and facilitators associated with varenicline adherence were extracted and defined according to the TDF, version 2 [27]. For studies that aimed at improving varenicline adherence, we used BCTTv1 [15] to extract data on the components of the intervention (active ingredients).

### Methodological quality assessment

We used the Joanna Briggs Institute’s (JBI) Critical Appraisal Tools [28] to assess the quality of randomized controlled trials (RCTs), quasi-experimental, analytical cross-sectional, case–control, cohort, and qualitative studies, and the Mixed Methods Appraisal Tool (MMAT) version 2018 [29] to assess the quality of mixed methods studies. For studies using the JBI Critical Appraisal Tool, an overall score was calculated based on the percentage of “Yes” answered, and questions were excluded from the overall score if “Not applicable” was answered. Studies with an overall score of 70% and above were deemed low risk of bias, studies with a score between 40 and 70% were deemed moderate risk of bias, and studies with a score of 40% and below were deemed high risk of bias [30]. Secondary and pooled analyses were assessed using the RCT checklist, and reference was made to the parent study. Since the use of an overall score to determine the quality of a study is not recommended for the MMAT, a detailed presentation of the quality assessment was provided for all included studies using the methodological quality criteria from the MMAT to determine whether it was of high, moderate, or low risk of bias [31].

The quality assessment was performed by two reviewers. One reviewer rated all included studies using the quality assessment form and a second reviewer verified the appraisal made by the first reviewer [20]. Conflicts were resolved by a third reviewer or by consensus.

### Data synthesis

We used a narrative synthesis of the included studies [20] to summarize the barriers and facilitators to varenicline adherence and the active ingredients in interventions that aim to help people adhere to varenicline. Barriers and facilitators were coded based on the 14 domains of the TDF (version 2). Active ingredients were coded based on the 16 groups of the BCTTv1. All reviewers were trained to code using the TDF and BCTTv1 (http://www.bct-taxonomy.com/). Discrepancies in coding were resolved by consensus or by an expert in TDF and BCTs (NM).

In order to understand which BCTs helped with adherence, we categorized the interventions into three simple categories: ‘effective’, ‘mixed results’ or ‘ineffective’. An intervention was categorized as ‘effective’ when improvements to medication adherence were reported to be statistically significant compared to the control group or baseline measures. Interventions were categorized as having ‘mixed results’ when the BCTs increased the participants’ knowledge, skills, or motivation but showed no sign of improving medication adherence. Interventions were categorized as ‘ineffective’ when the intervention did not significantly improve medication adherence compared to the control group or baseline measure.

Studies with low and moderate risk of bias were used to examine barriers and facilitators to varenicline adherence. In contrast, studies with high risk of bias were only used to confirm the patterns identified.

## Results

**Fig. 1:**
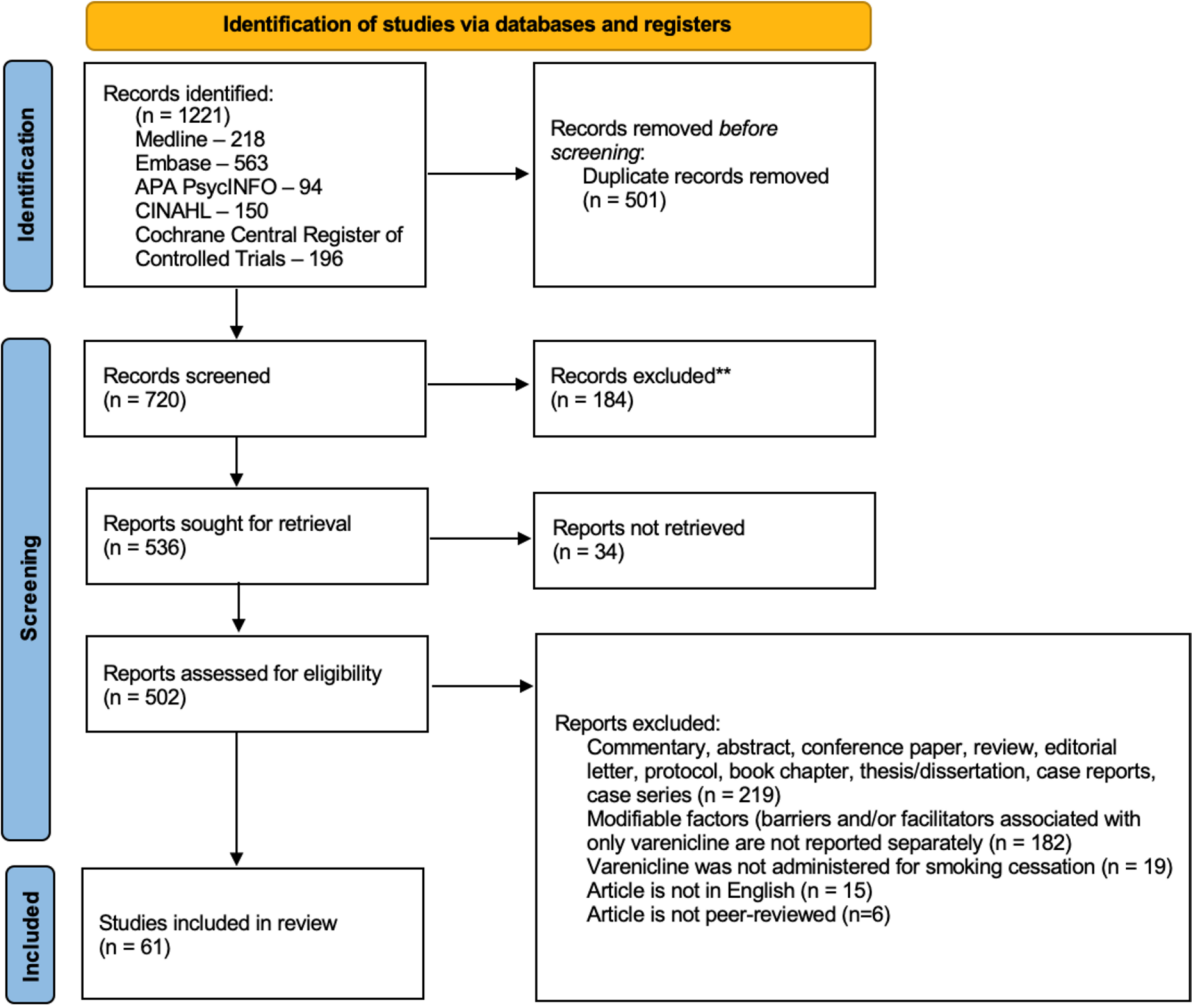
PRISMA flow diagram detailing the identification, screening, and inclusion of studies in the rapid review

A total of 1,221 titles were identified through the database searches; 61 met the eligibility criteria (see Fig. 1). Of these 61 studies, nine reported BCTs used to help participants adhere to varenicline.

### Study characteristics

Most of the studies included in this review consisted of RCTs (*n* = 38) [32–69], followed by cohort (*n* = 17) [70–86], cross-sectional (*n* = 3) [87–89], quasi-experimental (*n* = 3) [90–92], and qualitative (*n* = 1) [93] studies, and one study (*n* = 1) [53] was a mediation analysis that examined an observational study and RCT. The majority of studies had a low to moderate risk of bias [27 studies were of low risk of bias [37–39, 42, 44, 47–56, 61, 65, 71–73, 75, 83, 84, 86, 88, 92, 93]; 28 studies were of moderate risk [32, 33, 35, 40, 41, 43, 45, 46, 59, 60, 62–64, 66–70, 77–82, 85, 87, 89, 90]; and six studies were of high risk of bias [36, 57, 58, 74, 76, 91]]. Studies were conducted in a multitude of countries across all continents except Antarctica.

All studies focused on adult populations, and most focused on the general public (*n* = 43) [32, 33, 35, 37, 39–45, 48–53, 55, 59, 60, 62, 63, 65, 67, 69–74, 76, 77, 79–81, 83, 85–88, 90, 91, 93]. A few studies investigated specific patient populations: cancer (*n* = 3) [34, 36, 82]; chronic obstructive pulmonary disease (*n* = 3) [61, 75, 78]; human immunodeficiency viruses (*n* = 3) [54, 58, 66]; psychiatric conditions (*n* = 2) [46, 84]; and people undergoing substance use disorder treatment, including methadone treatment (*n* = 2) [47, 56]. There was a fairly even gender split among participants in the included studies, although 20 studies reported 30% or fewer female participants [71, 72, 38, 39, 77–79, 46, 48, 80, 81, 89, 54, 84, 55, 58, 92, 65, 66, 86]. Table 1 provides a summary of the included studies.

**Table 1:** Descriptive summary of included studies

Study (Author, Year, Reference)	Country of Study	Study Design, Sample Size, and Population	Percent Female Age (mean ± SD)	Definition of Adherence	Risk of Bias (Low, Moderate, or High)	Summary of Intervention
Aubin et al., 2008 [32]	Belgium; France; The Netherlands; United Kingdom; United States	RCT (*n* = 757); General population	Female = 50.8% Age = 42.9 ± 11.27**	NA	Moderate	52-week, open-label, randomized, multicenter, phase 3 trial comparing varenicline to NRT for smoking cessation. Participants were randomly assigned to varenicline uptitrated to 1 mg twice daily for 12 weeks or NRT (21 mg/day decreasing to 7 mg/day) for 10 weeks, while non-treatment follow-up continued until week 52
Balmford et al., 2011 [87]	Canada; United Kingdom; United States; Australia	Cross-Sectional (*n* = 1219); General population	Female = 60.5% Age = 45.5 ± 13.0	Completing the full course of 8 weeks of treatment	Moderate	ITC Four-Country Survey data was used in this study to (a) examine the prevalence and reasons for premature discontinuation of stop-smoking medications, including prescription-only medications, as well as (b) determine whether smokers or recent quitters that had taken medication within the past year differed by their type of medication used, their duration of use, and their source. (prescription or over the counter)
Bolliger et al., 2011 [33]	Brazil; Venezuela; Mexico; Columbia; Costa Rica; South Africa; Egypt; United Arab Emirates; Lebanon; Saudi Arabia; Jordan	RCT (*n* = 588); General population	Female = 39.6% Age = 43.4 ± 10.8	NA	Moderate	This multinational, randomized, double-blind, placebo-controlled trial based in Latin America, Africa, and the Middle East, tested varenicline for efficacy and tolerability among participants randomized (2:1) to varenicline 1 mg or placebo twice a day for 12 weeks, followed by 12 weeks of non-treatment follow-up and brief smoking cessation counselling
Boudrez et al., 2011 [70]	Belgium; Greece; Hungary; Slovenia	Cohort (*n* = 551);General population	Female = 46.5% Age = 45.8	NA	Moderate	In this 12-week prospective, observational, non-comparative trial, participants began treatment two weeks prior to their self-determined quit date while smoking abstinence rates and the safety of varenicline treatment were examined
Catz et al., 2011 [35]	United States	RCT (Secondary Analysis) (*n* = 1161); General population	Female = 66.8% Age = 47.3 ± 10.9	Taking at least 80% of prescribed varenicline.Good adherence was also defined as having an MAQ score of (M = 3.2, SD 0.8) at 21 days and a score of (M = 3.0, SD = 0.9) at 12 weeks after the participants quit date	Moderate	Smokers from a large health plan participated in this secondary analysis of the COMPASS Smoking Cessation Intervention Trial that aimed to determine the extent to which prescribed medications are taken by smokers, the impact this has on their cessation outcomes, and which factors may affect adherence. Participants were randomly assigned to receive different methods of cessation counselling combined with a 28-day supply of varenicline by mail with up to two refills and completed telephone surveys at baseline, 21 days, 12 weeks, and six months after setting a quit date
Chu et al., 2020 [71]	China	Cohort (*n* = 222); General population	Female = 3.2% Age = NA	NA	Low	To examine the safety of varenicline treatment between taxi-drivers and non-taxi-drivers, and the influence of treatment-related adverse events on driving behaviour in the taxi-driver group, this observational cohort study provided varenicline for up to 12 weeks and five standardized counselling sessions to both groups
Crawford et al., 2019 [36]	United States	RCT (Analysis) (*n* = 207);Cancer patients	Female = 50.7% Age = 58.48 ± 9.44	Varenicline adherence was defined as taking at least 80% of study medication across 12 weeks	High	Data collected from cancer patients receiving 12 weeks of open-label varenicline and counselling sessions through a clinical trial of varenicline for tobacco dependence in this population was used to examine (1) the interplay between self-reported varenicline adherence and verified smoking cessation as well as (2) correlates of varenicline adherence such as demographic and disease-related information, changes in cognition, affect, withdrawal, smoking reinforcement, and medication side effects
Ebbert et al., 2010 [72]	United States	Cohort (*n* = 20); General population	Female = 0% Age = 42.8 ± 11.7	NA	Low	As a strategy to reduce the use of tobacco among smokeless tobacco users who were not interested in quitting, this pilot study obtained preliminary data on the use of varenicline for 12 weeks
Ebbert et al., 2021 [37]	United States; Argentina; Australia; Brazil; Bulgaria; Canada; Chile; Denmark; Finland; Germany; Mexico; New Zealand; Russian Federation; Slovakia; South Africa; Spain	RCT (Post Hoc Analysis) (*n* = 8058);General population	Female = 56% Age = 46.5 ± 12.34	NA	Low	This multinational, multicenter, post-hoc analysis of the incidence, severity, and clinical trajectory of commonly reported adverse events following the use of smoking cessation pharmacotherapies from a large, phase 4, double-blind, randomized, triple-dummy, placebo-controlled trial (EAGLES) included smokers with and without psychiatric disorders, with treatments consisting of varenicline, bupropion sustained-release, and nicotine patch with tapering
Eisenberg et al., 2016 [38]	Canada; United States	RCT (*n* = 302); Patients with acute coronary syndrome	Female = 25% Age = 55 ± 9	NA	Low	This multicenter, double-blind, randomized, placebo-controlled trial evaluated the efficacy of varenicline administered in-hospital for smoking cessation based on point-prevalence smoking abstinence at 24 weeks, by providing hospitalized patients diagnosed with acute coronary syndrome with low-intensity counselling and randomizing them to varenicline or placebo for 12 weeks
Etter et al., 2013 [88]	France; Switzerland; United States; Canada; United Kingdom; Belgium; Over 30 other countries	Cross-Sectional (*n* = 1080); General population	Female = 69.5% Age = 43	NA	Low	Using an Internet survey, a French/English smoking cessation website (2008—2010) examined the use, compliance, and preferences for NRT, varenicline, and bupropion
Fagerström et al., 2010 [39]	Norway; Sweden	RCT (*n* = 431); General population	Female = 10.7% Age = 43.9 ± 12	NA	Low	An evaluation of the efficacy and safety of varenicline through a double-blind, placebo-controlled, parallel-group, multicentre, randomly controlled study was conducted in medical clinics, to assist smokeless tobacco users in quitting within four weeks, with the primary endpoint being four weeks of continuous abstinence at the conclusion of the treatment
Fouz-Rosón et al., 2017 [40]	Spain	RCT (*n* = 484); General population	Female = 40.5% Age = 50.67 ± 10.77	Taking 90% or more of the varenicline tablets	Moderate	In this open-label, randomized, parallel-group controlled trial, participants were randomized to 1 mg versus 0.5 mg and received behavioural support (baseline visit plus six follow-ups within one year) and one-year, continuous self-reported abstinence was biomedically verified
Gong et al., 2016 [41]	United States	RCT (*n* = 1002); General population	Female = 58.7% Age = 50.4 ± 11.1	NA	Moderate	An enhanced pharmacy care (EPC) program in which pharmacists provide personalized telephone counselling to support prescription smoking cessation medication was compared to usual care in order to assess the efficacy of the program. The primary outcome of the study was 1-week point prevalence at week 12. Participants were randomly assigned three telephone counselling sessions provided by specialist pharmacists at the beginning of the study or usual care with no counselling sessions
Gordon et al., 2017 [93]	United States	Qualitative (*n* = 5); General population	Female = 100% Age = 37.4	NA	Low	A prototype mobile health application for iOS was developed to assist smokers taking varenicline as part of their treatment of tobacco dependence in adhering to their medication regimen. Three phases were involved in developing the app, during which (1) input was received from consultants, focus groups, and user testing; (2) the feasibility of the app was assessed; and (3) a barcode scanner was developed and tested
Grassi et al., 2011 [73]	Italy	Cohort (*n* = 112); General population	Female = 54.2% Age = 51.14 ± 10.7**	NA	Low	The smoking cessation rate associated with 6 weeks of group counselling therapy given alone or in conjunction with 12 weeks of varenicline was investigated in a group of smokers
Gray et al., 2020 [42]	Canada; Georgia; South Korea; Russia; Taiwan; United States	RCT (*n* = 312); General population	Female = 36.2% Age = 15.9 ± 1.8**	Participants were considered non-adherent for the week if adherence was lower than 80%; participants were considered non adherent for the study if adherence was lower than 80% for the entire study treatment period	Low	This randomized, placebo-controlled trial evaluated the efficacy and tolerability of varenicline for smoking cessation in treatment-seeking adolescents at 57 outpatient centers. Participants were randomly assigned in a 1:1:1 ratio to varenicline at high doses, low doses, or placebo for 12 weeks, followed by 40 additional weeks of developmentally tailored smoking cessation counselling by trained counsellors (10 min per session)
Halperin et al., 2009 [43]	United States	RCT (*n* = 1018); General population	Female = 67% Age = 48 ± 11	NA	Moderate	This RCT compared three types of behavioural support (phone, web, and phone + web) for smoking cessation and examined symptoms, non-smoking rates, and medication usage among smokers undergoing varenicline treatment
Harrison-Woolrych et al., 2010 [74]	New Zealand	Cohort (*n* = 3415); General population	Female = 52% Age = NA	NA	High	This study evaluated varenicline's utilization and effectiveness during the first year of its marketing in New Zealand and compared it with the dosing instructions outlined in the product information through an analysis of dispensing records, including patient characteristics and usage patterns
Hays et al., 2010 [44]	United States	RCT (Pooled Analysis) (*n* = 2045); General population	Female = 44% Age = 42.9 ± 11.6	Taking at minimum 80% of prescribed varenicline	Low	This study assessed adherence to tobacco dependence treatment, the relationship between adherence and abstinence, and predictors of adherence, using pooled results from two RCT’s in which adult smokers were randomly assigned to 12 weeks of treatment and took at least 1 dose of the assigned medication (varenicline, bupropion, or placebo) in conjunction with a 12-week brief smoking cessation counselling
Hernández Zenteno et al., 2018 [75]	Mexico	Cohort (*n* = 94); People with chronic obstructive pulmonary disease	Female = 50% Age = 52 ± 12	NA	Low	In this observational study, smoking cessation rates were compared in patients with chronic obstructive pulmonary disease versus non-obstructed smokers following varenicline treatment for 12 weeks paired with cognitive behavioural therapy
Heydari et al., 2017 [90]	Iran	Quasi-Experimental (*n* = 227); General population	Female = 41.6% Age = 43.1	NA	Moderate	This quasi-experimental study aimed to determine the duration of Champix use based on its cost by examining smokers attending the Tanaffos Smoking Cessation Clinic in Tehran, Iran, after consulting with a physician and beginning treatment with Champix (at least 10 weeks, including 2 weeks for starter pack 0.5 and 1 mg, 4 weeks for the first maintenance pack 1 mg, and 4 weeks for the second maintenance pack 1 mg). Following the intervention, patients were monitored by telephone as well as by regular clinic visits
Hodgkin et al., 2013 [76]	United States	Cohort (Analysis) (*n* = 291); General population	Female = 55.7% Age = 52.5 ± 10.7	NA	High	Using a patient-centred approach, this observational report describes the outcome rate of treatment at St. Helena Center for a Smoke-Free Life in terms of any combination of short and long-acting tobacco dependence medications and group behavioural counselling, interactive educational sessions, regular exercise and healthy nutrition for patients in the program
Jiménez-Ruiz et al., 2013 [77]	Spain, Uruguay	Cohort (*n* = 73);General population	Female = 25% Age = 51.47 ± 14.34	NA	Moderate	The study presents clinical results of varenicline treatment in consecutive smokers who did not respond to the standard dose and had previously received smoking cessation services in the form of ten individual sessions of behavioural therapy and varenicline at a dose of three mg per day
Jiménez-Ruiz et al., 2017 [78]	Spain	Cohort (*n* = 79); People with chronic obstructive pulmonary disease	Female = 24.1% Age = 60.6 ± 8	NA	Moderate	In this post-authorization, open-label prospective follow-up study, varenicline was evaluated for its effectiveness in treating patients with severe or very severe chronic obstructive pulmonary disease by extending the usual 12-week treatment period to 24 weeks, while ensuring continuous abstinence between weeks 9 and 24 of the treatment
Jorenby et al., 2006 [45]	United States	RCT (*n* = 1027); General population	Female = 42.2% Age = 43.3 ± 11.64**	NA	Moderate	This randomized, double-blind, multi-center placebo-controlled trial on adult smokers aimed to determine the efficacy and safety of varenicline titrated to 1 mg twice daily compared with placebo or sustained-release bupropion, as well as weekly brief smoking cessation counselling for 12 weeks with follow-up at week 52
Jung et al., 2010 [79]	South Korea	Cohort (*n* = 217); General population	Female = 7.8% Age = 52	NA	Moderate	An evaluation of the effectiveness of varenicline for smoking cessation was conducted in a pulmonary clinic at a university-affiliated hospital in South Korea by retrospectively reviewing medical records, screening smoking status via telephone interview, and prescribing varenicline after brief, standardized, individual counselling. The primary outcome was 4-week continuous abstinence from smoking between 9 and 12 weeks
Meszaros et al., 2013 [46]	United States	RCT (*n* = 10); Psychiatric conditions	Female = 30% Age = 43 ± 7	NA	Moderate	A double-blind, randomized, placebo-controlled study of outpatients with schizophrenia or schizoaffective disorder and concurrent alcohol and nicotine dependence was conducted to determine if varenicline in conjunction with weekly individual motivational interviewing was a safe and effective treatment method
Nahvi et al., 2020 [47]	United States	RCT (*n* = 100); Individuals in substance use disorder treatment	Female = 44% Age = 49	Adherence was measured by pill count and remaining pills were counted weekly	Low	This multicenter, parallel-group two-arm randomized controlled trial aimed to evaluate the efficacy of directly observed therapy on varenicline adherence and smoking cessation rates among smokers with opioid use disorder who were receiving methadone treatment by randomly assigning them to 12 weeks of varenicline, either directly observed or via unsupervised self-administered treatment, with the primary outcome being adherence based on pill count
Nakamura et al., 2017 [48]	Japan	RCT (Subpopulation Analysis) (*n* = 210); General population	Female = 30% Age = 42.5 ± 12.4***	NA	Low	This prospective analysis of the Japanese subpopulation of the varenicline Reduce to Quit Trial was conducted to determine whether the results were consistent with that of the full study population. Patients were prescribed varenicline or placebo for 24 weeks (12 weeks smoking reduction phase and 12 weeks smoking abstinence phase) with the primary efficacy endpoint being continuous abstinence from weeks 15 to 24, followed by a 28-week follow-up phase
Niaura et al., 2008 [49]	United States	RCT (*n* = 320); General population	Female = 48.1% Age = 41.5 ± 11.3	. Adherence was measured by counting any returned varenicline blister packs weekly	Low	The multicenter, randomized, double-blind, placebo-controlled study used varenicline (fixed dose at Week 1: titrated from 0.5 to 1.0 mg per day, then self-regulated flexible schedule at Weeks 2–12: 0.5 to 2.0 mg per day) or placebo for smokers who had been healthy and motivated to quit smoking, followed by a 40-week, double-blind, nontreatment follow-up to determine whether self-regulated flexible dosing with varenicline tartrate was safe and effective for smoking cessation
Nides et al., 2006 [50]	United States	RCT (*n* = 638);General population	Female = 51.8%***, Age = 41.8 ± 10.4**	NA	Low	A phase 2, multicenter, randomized, double-blind, placebo-controlled study evaluating 3 varenicline doses was conducted in healthy smokers who were randomized to receive either varenicline tartrate (0.3 mg once daily, 1.0 mg once daily or 1.0 mg twice daily, for 6 weeks plus placebo for 1 week), or sustained-release bupropion hydrochloride (150 mg twice daily for 7 weeks), or placebo (7 weeks); while bupropion hydrochloride was included as an active control
Nides et al., 2008 [51]	United States	RCT (Pooled Analysis) (*n* = 2051); General population	Female = 44.1% Age = 42.8 ± 11.6***	NA	Low	In two randomized, placebo-controlled trials, smokers received varenicline, bupropion SR, or placebo for 12 weeks, followed by a 40-week non-treatment follow-up with the aim to determine whether varenicline functions as an effective smoking cessation agent compared to bupropion SR and placebo, as well as whether factors typically associated with abstinence can influence varenicline's efficacy versus placebo, as measured by the week 9–12 continuous abstinence rate.
Ock et al., 2022 [80]	Korea	Cohort (*n* = 3719);General population	Female = 9.1% Age = 49.4 ± 11.7	NA	Moderate	This study performed post-marketing surveillance on Korean smokers over a 12-week period by administering varenicline. A 7-day point prevalence of smoking cessation during the treatment course is used to assess the safety and effectiveness of varenicline in usual medical practice in South Korea
Park et al., 2011a [81]	Philippines	Cohort (*n* = 330);General population	Female = 16.7% Age = 46 ± 12.8	NA	Moderate	In this surveillance study, adult Filipino smokers who had been prescribed varenicline were monitored for a period of 12 weeks to assess the safety and efficacy of varenicline
Park et al., 2011b [82]	United States	Cohort (*n* = 49); Cancer patients	Female = 59.2% Age = 57.7 ± 12.4	NA	Moderate	This study aimed to determine whether a 12-week program that combines smoking cessation counselling with varenicline is feasible and possibly effective in patients with thoracic cancer or suspected thoracic malignancy. At the end of treatment, seven-day point prevalence rates of tobacco abstinence were compared with a control group receiving usual care
Peng et al., 2017 [52]	United States; Canada	RCT (Secondary Analysis) (*n* = 376);General population	Female = 44.9% Age = 43.5 ± 11.5	Adherence was measured by calculating pills taken divided by number of prescribed pills. As well as a salivary varenicline concentration of 4.7 ng/ml (CI 95%: 4.06–5.36)	Low	This secondary analysis of data from a randomized, placebo-controlled clinical trial compared the efficacy of varenicline and transdermal nicotine for treating nicotine dependence among slow and fast metabolizers of nicotine, and to evaluate predictors of adherence to varenicline. Comparisons were made between the number of pills collected over 4 different time periods and varenicline salivary levels after 2 weeks of treatment
Peng et al., 2020 [53]	United States (Study 1 and 2); Canada (Study 2)	RCT and Cohort (Mediation Analysis) (Sample 1 *n* = 449; Sample 2 *n* = 421);General population	Female = 52% Age = 42.2 ± 11.7	Varenicline adherence was assessed through LC–MS/MS by measuring varenicline concentrations in saliva or plasma samples of participants	Low	This study recruited treatment-seeking smokers that received varenicline from two smoking cessation clinical trials, to examine how nausea might affect smoking cessation outcomes mediated by adherence at several sites within North America. Sample 1 received 12 weeks of varenicline treatment combined with smoking cessation counselling, while Sample 2 was given 12 weeks of varenicline treatment combined with behavioural counselling
Pozzi et al., 2015 [83]	Italy	Cohort (*n* = 187); General population	Female = 37.4% Age = 55.9 ± 4.6	NA	Low	As part of the Multi-centric Italian Lung Detection Trial, this prospective analysis study examined the biochemically verified 1-year continuous abstinence rate of persistent smokers who were randomized to receive varenicline to quit smoking as well as cognitive behavioural counselling
Purvis et al., 2009 [89]	United States	Cross-Sectional (*n* = 50);Veteran population	Female = 14% Age = 60	NA	Moderate	This evaluation of a prospective performance measure involving veterans initiated on varenicline was conducted to examine varenicline's safety profile with regard to psychiatric symptoms, its effectiveness, as well as associations between certain baseline demographics and success rates. Throughout treatment and at week 12, patients were contacted via telephone for follow-up, during which they were asked about their smoking history and provided with medication counselling, with the primary endpoint being cessation between weeks 9 and 12
Quinn et al., 2020 [54]	United States	RCT (Analysis) (*n* = 89); Patients with human immunodeficiency viruses	Female = 28.1% Age = 48.7 ± 10.1	Taking at minimum 80% of prescribed varenicline	Low	This study conducted secondary analyses based on a randomized placebo-controlled trial of varenicline for smoking among HIV/AIDS patients in order to examine the relationship between varenicline adherence based on pill count and smoking cessation after treatment, along with correlates of varenicline adherence. Participants received varenicline in conjunction with six standardized, guideline-compliant smoking cessation counselling sessions
Raich et al., 2016 [84]	Spain	Cohort (*n* = 90);Psychiatric conditions	Female = 28.6% Age = 44.8 ± 10.1	NA	Low	In this longitudinal, multicenter study, three groups of patients with psychiatric disorders (psychotic disorder, alcohol dependence disorder, methadone maintenance treatment addicts) underwent 12 weeks of cognitive-behavioural psychological treatment and pharmacological treatment with varenicline in order to determine if varenicline was safe for smoking cessation
Ramon et al., 2009 [91]	Spain	Quasi-Experimental (*n* = 264);General population	Female = 41.3% Age = 43.7 ± 10.1	NA	High	To evaluate the effectiveness of varenicline paired with cognitive behavioural interventions aimed at developing a specific action plan and alternative behaviours, this interventional study was conducted among motivated smokers attending two smoking cessation clinics
Rigotti et al., 2010 [55]	United States; Argentina; Australia; Brazil; Canada; Czech; Denmark; France; Germany; Greece; South Korea; Mexico; The Netherlands; Taiwan; United Kingdom	RCT (*n* = 714); General population	Female = 21.3% Age = 56.5 ± 8.5***	NA	Low	As part of a multicenter, randomized, double-blind, placebo-controlled trial, smokers with stable cardiovascular disease were randomized to varenicline for 12 weeks along with smoking cessation counselling and monitored for 52 weeks in order to evaluate the safety and efficacy of varenicline for quitting smoking in this population
Rohsenow et al., 2017 [56]	United States	RCT (*n* = 137); Individuals in substance use disorder treatment	Female = 47% Age = 39.6 ± 10	Adherence was measured by counting any returned patches, MEMSCap data, and the percentage of capsules taken	Low	Adult smokers in substance use disorder treatment who had been substance abstinent for less than 12 months were recruited to participate in this double-blind placebo-controlled randomized trial stratified by major depressive disorder, gender, and nicotine dependence, and were given either varenicline or NRT for 12 weeks, with the primary outcome being verified 7-day smoking abstinence at 3 months
Scoville et al., 2020 [57]	United States	RCT (Observational Study *n* = 1098; RCT *n* = 32); People with Crohn’s disease	Female = 53.1% Age = 44 ± 11.1	NA	High	This observational study examined the prevalence of smoking among patients with Crohn's disease and evaluated how they perceive personalized metabolism-informed care (MIC). NRT was recommended for slow nicotine metabolizers, whereas non-nicotine-based methods were recommended for normal nicotine metabolizers. The primary outcomes included intervention satisfaction and medication choice match rates based on nicotine metabolite ratios
Shelley et al., 2015 [58]	United States	RCT (Analysis) (*n* = 158); Patients with human immunodeficiency viruses	Female = 13.4% Age = 46.94 ± 9.75	Taking 80% or more of the prescribed medication for the last 4 weeks at each one month follow up visit, confirmed through pill count	High	Participants from three HIV/AIDS care centers in New York City were recruited and enrolled in a three-arm randomized controlled pilot study. They were randomized to receive varenicline alone or combined with two adherence-focused support options, twice daily text messages or text messages plus seven counselling sessions delivered on cell phones, this report analyzes correlates of adherence to varenicline in this population
Skelton et al., 2022 [92]	Australia	Quasi-Experimental (*n* = 20);Homeless population	Female = 0% Age = 44 ± 9.3	Taking varenicline for at least 80% of the days prescribed	Low	This single-group pilot study aimed to determine whether varenicline in combination with NRT and motivational interviewing could be effectively administered to adult male smokers attending a clinic in a homeless hostel
Stein et al., 2013 [59]	United States	RCT (*n* = 315); General population	Female = 50.5% Age = 39.9 ± 9.7	Adhernece was defined as the percentage of days the participants disclosed using the medication during the first 30 days of follow-up	Moderate	Using a three-group randomized design, the efficacy of varenicline vs. placebo compared to NRT (combination of nicotine patch and libitum nicotine rescue) and minimal behavioral intervention at baseline (NCI’s 5A’s) was evaluated in a 6-month treatment for smoking cessation among methadone-maintained smokers from nine treatment centers in southern New England
Swan et al., 2012 [60]	United States	RCT (Analysis) (*n* = 397); General population	Female = 68% Age = 49.2	NA	Moderate	The association between common and rare sequence variants in 10 nicotinic acetylcholine receptor subunit genes was evaluated and the severity of nausea 21 days after initiating the standard varenicline regimen for smoking cessation, and was an analysis of data based on participants from a randomized clinical effectiveness trial with complete clinical and DNA resequencing data in which participants were randomized to one of three modes of delivery of behavioral treatment: telephone-based, Web-based, or a combined telephone/Web-based intervention and were prescribed a standard 12-week course of varenicline
Tashkin et al., 2011 [61]	United States; Spain; France; Italy	RCT (*n* = 504);People with chronic obstructive pulmonary disease	Female = 37.7% Age = 57.2 ± 9.1***	NA	Low	Data from a randomized clinical effectiveness trial with complete clinical and DNA resequencing data was analysed. Participants were assigned to one of three delivery methods of behavioral treatment: telephone, web-based, or a combined telephone/web-based intervention and were prescribed varenicline for 12 weeks. Ten nicotinic acetylcholine receptor subunit genes were analysed for the association between common and rare variants and nausea 21 days after starting varenicline therapy
Tonstad et al., 2006a [63]	United States; Canada; Czech Republic; Denmark; Norway; Sweden; United Kingdom	RCT (*n* = 1210);General population	Female = 50.7% Age = 44.6 ± 10.6***	NA	Moderate	An RCT was conducted at multiple medical clinics in seven countries with follow-up until 52 weeks after baseline to determine whether smokers who quit after 12 weeks of varenicline treatment maintain higher rates of continuous abstinence. The study participants were randomly assigned to receive either varenicline or placebo for an additional 12 weeks. The primary outcome was sustained abstinence between weeks 13 to 24 and between weeks 13 and 52
Tonstad et al., 2006b [62]	United States; Canada; Czech Republic; Denmark; Norway; Sweden; United Kingdom	RCT (Study 1 *n* = 1025; Study 2 *n* = 1027); General population	Female = 48.1%*** Age = 43.9 ± 11.0***	NA	Moderate	During two randomized, double-blind studies, smokers who were motivated to quit were provided with brief smoking cessation counselling and varenicline (1 mg twice daily) in comparison to treatment with bupropion (150 mg twice a day) or a matching placebo for 12 weeks, followed by a 40-week observation period without treatment. A third study looked at maintaining abstinence in smokers by administering open-label varenicline (1 mg twice a day) for 12 weeks. Those who did not smoke during the last week of treatment were randomized to varenicline or placebo for an additional 12 weeks, followed by a 28-week observation period without treatment
Tonstad et al., 2017 [64]	United States, France, Italy, Spain, Argentina, Australia, Brazil, Canada, Czech Republic, Denmark, Germany, Greece, South Korea, Mexico, Netherlands, Taiwan, United Kingdom, Japan, China, Singapore, Thailand, Columbia, Costa Rica, Egypt, Jordan, Lebanon, Saudi Arabia, South Africa, United Arab Emirates, Venezuela, Hungary, Norway, Sweden, Bosnia and Herzegovina, Croatia, Romania, Russian Federation, Belgium	RCT (Pooled Analysis) (*n* = 6771); People with diabetes	Female = 36%*** Age = 45.6 ± 12.1***	Adherence was determined through 2 and 4 week visits and overall adherence wasdefined as the percentage of days a particpants used their prescribed medication during the first 30 days	Moderate	This retrospective pooled analysis included data collected from smokers with diabetes who participated in 15 double-blind, randomized, placebo-controlled studies to assess varenicline's efficacy and safety. Participants were given varenicline (1 mg twice a day) or a placebo for 12 weeks, and continuous abstinence rates were evaluated for weeks 9–12, 9–24 and 9–5
Tsai et al., 2007 [65]	South Korea; Taiwan	RCT (*n* = 250);General population	Female = 11.2% Age = 40.3 ± 10.2***	NAJ47	Low	In Korea and Taiwan, a 12-week treatment, 12-week follow-up trial was conducted at five sites with smokers receiving smoking cessation counseling and being randomly assigned varenicline (1 mg twice a day, titrated in the first week) or placebo. The primary endpoint of the study was continuous abstinence rate during the final four weeks of treatment
Tseng et al., 2017 [66]	United States	RCT (*n* = 158); Patients with human immunodeficiency viruses	Female = 18.4% Age = 46.79 ± 9.83)	Taking at least 80% of their assigned varenicline	Moderate	The goal of this RCT was to promote varenicline adherence and smoking abstinence among smokers living with HIV by combining text messaging and telephone counseling. Participants were recruited from three HIV care centers in New York City and randomly assigned to 2-weeks of varenicline either alone or in conjunction with text message support or text message and adherence-focused motivational and behavioral therapy delivered by cell phone
Tulloch et al., 2016 [67]	Canada	RCT (*n* = 737);General population	Female = 46.6% Age = 48.61 ± 10.8	NA	Moderate	An RCT was conducted in which smokers with and without medical or psychiatric comorbidities were randomly assigned to one of three treatment conditions: The NRT group was given 10 weeks of patches; the NRT + group received patches and gum or inhaler for up to 22 weeks; and the varenicline group was given varenicline for up to 24 weeks. In addition, each participant received six standardized 15-min counselling sessions on smoking cessation, with continuous abstinence rates from weeks 5 through 52 as the primary outcome
Van Boven et al., 2016 [85]	The Netherlands	Cohort (*n* = 4241);General population	Female = 47.9% Age = 45.73 ± 13.11 **	Taking at least 80% of the prescribed tablets	Moderate	A retrospective dispensing database analysis of real-world observational data from the Netherlands was conducted in order to examine the impact of reimbursement of smoking cessation treatments on patients' use and adherence to pharmacological assisted smoking cessation. Patients who were dispensed bupropion or varenicline for the first time in community pharmacies were assessed for use and adherence
Walker et al., 2021 [68]	New Zealand	RCT (*n* = 679); Indigenous Māori or whhanauānau population	Female = 69.7%*** Age = 43.3 ± 11.9***	Taking 80% or more of varenicline 3 months after participants quit date	Moderate	This open-label, randomized, community-based non-inferiority trial was conducted to determine whether cytisine was at least as effective as varenicline in supporting smoking abstinence for over 6 months among indigenous Māori or whhanauānau populations. Daily smokers were randomly assigned in a 1:1 ratio to receive either cytisine or varenicline for 12 weeks along with low-intensity cessation behavioural support. The primary outcome was continuous abstinence at 6 months
Wang et al., 2013 [86]	China; India; Philippines; Korea	Cohort (*n* = 1373); General population	Female = 6.8% Age = 45.2 ± 12.5	NA	Low	This multicenter, prospective, non-comparative, observational study examined the effectiveness and safety of varenicline in clinical practice among Asian adult smokers who wanted to quit smoking and agreed to take 1 mg of varenicline twice daily (after titration of 1 week) for 12 weeks after reaching a joint decision with the investigators
Williams et al., 2007 [69]	United States; Australia	RCT (*n* = 377); General population	Female = 50.1% Age = 48	NA	Moderate	In this multicenter, randomized, double-blind trial, adult smokers were randomized to receive varenicline 1mg twice daily or placebo for 52 weeks in order to assess the safety of long-term varenicline administration for smoking cessation. At each visit, brief counseling was provided, and vital signs, adverse events, and smoking status were recorded

### Barriers and facilitators – by Theoretical Domains

Most studies included in this review reported barriers as opposed to facilitators. Of the 61 studies, 51 studies [32, 33, 36–40, 42–57, 59–65, 67–69, 71, 73–78, 80–83, 86–92] only mentioned barriers, while four studies [41, 58, 66, 85] only mentioned facilitators, and six studies mentioned both barriers and facilitators [35, 37, 70, 79, 84, 93]. Definitions of the theoretical domains according to Atkins, et al. [94] can be found in Table 2.

**Table 2:** Barriers and facilitators to varenicline adherence according to the Theoretical Domains framework

Theoretical Domains Framework Theme	Influence (Barrier/Facilitator)	Study	Summary of Theme
**Knowledge** “An awareness of the existence of something” [94]	Facilitator (*n* = 3)	Gong et al. 2016, [41]; Shelley et al. 2015, [58]; Ebbert et al. 2021, [37]	*Facilitator* Knowledge about varenicline and providing information about the benefits of quitting smoking and medication adherence [57, 40] Providing ongoing support through education and information about the course of treatment and treatment expectations [36]
**Beliefs about capabilities** "Acceptance of the truth, reality or validity about an ability, talent or facility that a person can put to constructive use" [94]	Barrier (*n* = 10) Facilitator (*n* = 3)	Walker et al. 2021, [68]; Halperin et al. 2009, [43]; Harrison-Woolrych et al. 2010, [74]; Jung et al. 2010, [79]; Balmford et al. 2011, [87]; Grassi et al. 2011, [73]; Catz et al. 2011, [35]; Etter et al. 2013, [88]; Raich et al. 2016, [84]; Jiménez-Ruiz et al. 2017, [78] Catz et al. 2011, [35]; Shelley et al. 2015, [58]; Tseng et al. 2017, [66]	*Barrier* Perceived competence: Did not need to take medication anymore to abstain from smoking [72, 42, 73, 86, 34, 67]. Participants discontinued study medication because they did not think they needed it anymore after abstaining from smoking for 12 weeks [83]. Discontinued participation in study because participants were able to stop smoking before completing the study [77]. Could not stop smoking and relapsed [87] Self-efficacy: low will power made participants discontinue study medication [78]. Did not need to take medication anymore to abstain from smoking [34, 72] Beliefs: Did not need to take medication anymore to abstain from smoking [72] *Facilitator* Self-efficacy: higher self-efficacy encouraged varenicline adherence [34, 57, 65]
**Optimism** “The confidence that things will happen for the best or that desired goals will be attained” [94]	Barrier (*n* = 17)	Halperin et al. 2009, [43]; Bolliger et al. 2011, [33]; Ock et al. 2022, [80]; Nides et al. 2008, [51]; Nides et al. 2006, [50];Purvis et al. 2009, [89]; Rigotti et al. 2010, [55]; Harrison-Woolrych et al. 2010, [74]; Jung et al. 2010, [79]; Balmford et al. 2011, [87]; Grassi et al. 2011, [73]; Catz et al. 2011, [35];Park et al. 2011, [81]; Etter et al. 2013, [88]; Wang et al. 2013, [86]; Raich et al. 2016, [84]; Scoville et al. 2020, [57]	*Barrier* Pessimism- Discontinued use of varenicline due to relapsing back to smoking. Medication failed to make participants quit smoking [86, 83], participants perceived low efficacy, [32, 78, 49, 49, 79, 80, 54], discontinued varenicline because they thought it was not working [34, 72, 42], experiencing cravings and withdrawal symptoms, or relapsed back to smoking [87], lack of beneficial response to varenicline [73, 88, 56, 85]
**Beliefs about Consequences** “Acceptance of the truth, reality, or validity about outcomes of a behaviour in a given situation” [94]	Barrier (*n* = 53)	Balmford et al. 2011, [87]; Aubin et al. 2008, [32]; Bolliger et al. 2011, [33]; Boudrez et al. 2011, [70];Catz et al. 2011, [35]; Chu et al. 2020, [71]; Crawford et al. 2019, [36]; Ebbert et al. 2010, [72]; Ebbert et al. 2021, [37]; Eisenberg et al. 2016, [38]; Etter et al. 2013, [88]; Fagerström et al. 2010, [39]; Fouz-Rosón et al. 2017, [40]; Gordon et al. 2017, [93]; Grassi et al. 2011, [73]; Gray et al. 2020, [42]; Halperin et al. 2009, [43]; Harrison-Woolrych et al. 2010, [74]; Hays et al. 2010, [44]; Hernández Zenteno et al. 2018, [75]; Hodgkin et al. 2013, [76]; Jiménez-Ruiz et al. 2013, [77]; Jiménez-Ruiz et al. 2017, [78]; Jorenby et al. 2006, [45]; Jung et al. 2010, [79]; Meszaros et al. 2013, [46]; Nahvi et al. 2020, [47]; Nakamura et al. 2017, [48]; Niaura et al. 2008, [49]; Nides et al. 2006, [50]; Nides et al. 2008, [51]; Ock et al. 2022, [80]; Park et al. 2011, [81]; Park et al. 2011, [82]; Peng et al. 2020, [52]; Pozzi et al. 2015, [83]; Purvis et al. 2009, [89]; Quinn et al. 2020, [54]; Raich et al. 2016, [84]; Ramon et al. 2009, [91]; Rigotti et al. 2010, [55]; Rohsenow et al. 2017, [56]; Scoville et al. 2020, [57]; Stein et al. 2013, [59]; Swan et al. 2012, [60]; Tashkin et al. 2011, [61]; Tonstad et al. 2006a, [63]; Tonstad et al., 2006b, [62]; Tonstad et al. 2017, [64]; Tsai et al. 2007, [65]; Tulloch et al. 2016, [67]; Walker et al. 2021, [68]; Wang et al. 2013, [86]; Williams et al. 2007, [69]	*Barrier* Outcome expectancies: discontinued varenicline usage because participants would relapse due to medication not working [86]. Felt study medication was ineffective [56] Adverse effects- Nausea [31, 39, 72, 74–76, 44–46, 48–50, 80, 81, 51, 88, 90, 56, 59, 62, 64, 85, 61], headache [39, 72, 75, 46, 80, 88, 62, 64], vomiting [35, 42, 76, 45, 46, 64], insomnia/sleep problem [35, 38, 72, 75, 46, 53, 90, 55, 56, 62, 64, 85], abnormal dreams [72, 81, 88, 56, 85], constipation [46, 88, 64], gas [46, 64, 83], suicide ideation [45, 83, 31], depression [35, 42, 75, 90, 55, 62], fatigue [46, 62], dizziness [46, 85], irritability [45, 46, 56], anxiety [74, 55], nightmares [39, 55], rash [55, 64] Other AEs: random loss of consciousness throughout the day [38], dyspepsia [72], digestive intolerance [77], change in taste and appetite [46], agitation/aggression [81], seizure [55], distorted thoughts and agitation [56], hearing voices and mood disturbance [58], peritonitis/acute appendicitis and acute pyelonephritis [64], sever unstable angina [64], snoring [83], gastrointestinal problems [83], Other (AE was stated, but not specified): 22 studies [32, 69, 34, 70, 71, 36, 37, 87, 92, 41, 73, 78, 79, 47, 82, 54, 60, 63, 66–68, 61]
**Intentions** “A conscious decision to perform a behaviour or a resolve to act in a certain way” [94]	Barrier (*n* = 1)	Jiménez-Ruiz et al. 2017, [78]	*Barrier* Stages of change model: discontinuation of participation in study due to an unclear purpose to abstain from smoking [77]
**Goals** “Mental representations of outcomes or end states that an individual wants to achieve” [94]	Barrier (*n* = 1)	Raich et al. 2016, [84]	*Barrier* Goals (autonomous): Discontinuation of study participation due to low motivation and relapsing back to smoking [83]
**Memory, attention and decision processes** "The ability to retain information, focus selectively on aspects of the environment and choose between two or more alternatives" [94]	Barrier (*n* = 2)	Walker et al. 2021, [68]; Skelton et al. 2022, [92]	*Barrier* Memory: Forgetting to take medication [91, 67] Decision making: participants could not be bothered to pick up their medication and did not want to take the medication [67]
**Environmental context and resources** “Any circumstance of a person’s situation or environment that discourages or encourages the development of skills and abilities, independence, social competence and adaptive behaviour” [94]	Barrier (*n* = 9) Facilitator (*n* = 3)	Walker et al. 2021, [68]; Halperin et al. 2009, [43]; Heydari et al. 2017, [90]; Hernández Zenteno et al. 2018, [75]; Ock et al. 2022, [80]; Harrison-Woolrych et al. 2010, [74]; Jung et al. 2010, [79]; Peng et al. 2017, [52]; Jiménez-Ruiz et al. 2017, [78] Jung et al. 2010, [79]; Van Boven et al. 2016, [85]; Raich et al. 2016, [84]	*Barrier* Resources/material resources: participants discontinued study medication because they did not try to refill their prescription pill pack when it ran out [42]. Participants discontinued varenicline because it was too expensive for them [73, 74, 89, 78, 79]. Participants did not obtain the study drug because they could not make it to the pharmacy [67] Environmental stressors: participant discontinued varenicline due to situational factors causing them anxiety [77] Barriers and facilitators: the crave to smoke was significantly lower in participants who adhered to varenicline [51] *Facilitator* Barriers and facilitators: reducing the dosage of varenicline to 1 mg/day to decrease adverse effects and relevant psychiatric symptoms that appeared [83] Resources/material resources: low willpower caused regular clinic patients to discontinue varenicline compared to volunteer participants. Clinic patients potentially need better support systems to allow for varenicline adherence [78]. Participants adhered to their varenicline more frequently during reimbursement periods than non-reimbursement periods [84]
**Social influences** “Those interpersonal processes that can cause individuals to change their thoughts, feelings, or behaviours” [94]	Facilitator (*n* = 4)	Gong et al. 2016, [41]; Jung et al. 2010, [79]; Boudrez et al. 2011, [70]; Ebbert et al. 2021, [37]	*Facilitator* Social support: The relationship between a supportive and personalized counselor encouraged varenicline adherence and smoking abstinence [69]. Providing ongoing support through education and information about the course of treatment and treatment expectations [36]. Providing knowledge about varenicline and information about the positive effects of not smoking and medication adherence through counselling [40]. Low willpower caused regular clinic patients to discontinue varenicline use compared to volunteer participants, and thus, clinic patients may need better support systems to improve varenicline adherence [78]
**Behavioural regulation** “Anything aimed at managing or changing objectively observed or measured actions” [94]	Barrier (*n* = 3) Facilitator (*n* = 1)	Harrison-Woolrych et al. 2010, [74]; Jung et al. 2010, [79]; Park et al. 2011, [81] Gordon et al. 2017, [93]	*Barrier* Self-monitoring: participants who prematurely discontinued varenicline did so due to poor compliance [73, 80], and low will power [78] *Facilitator* Self-monitoring: having an accessible and easy to use app that reminded participants to take their medication [92]

‘Belief about consequences’ was the most common barrier to varenicline adherence, reported by 53 of the included studies. Moreover, 52 of these studies reported side effects as a barrier to adherence (27 of the studies were of low/moderate risk of bias). The most frequently reported adverse effects contributing to varenicline non-adherence were nausea (*n* = 23; 20 low/moderate risk of bias) [32, 40, 45–47, 49–51, 53, 57, 60, 62, 63, 65, 73, 75–77, 81, 82, 86, 89, 91]; insomnia/sleep problems (*n* = 12; 8 low/moderate risk of bias) [36, 39, 47, 54, 56, 57, 63, 65, 73, 76, 86, 91]; headache (*n* = 8; 7 low/moderate risk of bias) [40, 47, 63, 65, 73, 76, 81, 89]; depression (*n* = 7; 4 low/moderate risk of bias) [32, 36, 43, 56, 62, 76, 91]; vomiting (*n* = 6; 5 low/moderate risk of bias) [36, 43, 46, 47, 65, 77]; and abnormal dreaming (*n* = 5; 4 low/moderate risk of bias) [57, 73, 82, 86, 89].

Another frequently cited domain mentioned as a barrier to varenicline adherence was optimism/pessimism, which was reported in 17 studies (*n* = 17; 15 low/moderate risk of bias) [33, 35, 43, 50, 51, 55, 57, 73, 74, 79–81, 84, 86–89]. In these studies, participants discontinued their varenicline treatments as their confidence that varenicline would help them quit smoking diminished.

Ten studies (*n* = 10; 9 low/moderate risk of bias) [35, 43, 68, 73, 74, 78, 79, 84, 87, 88] mentioned beliefs about capabilities as a determinant of varenicline adherence. These studies reported perceived competence and/or low willpower as contributors to varenicline discontinuation.

Nine studies mentioned environmental context and resources (*n* = 9; 8 low/moderate risk of bias) [43, 52, 68, 74, 75, 78–80, 90] as a barrier to varenicline adherence. The cost of the medication and lack of access to pharmacies were the most frequent barriers coded under this domain.

Other less frequently reported barriers to varenicline adherence include: behavioural regulation (*n* = 3; 2 moderate risk of bias) [74, 79, 81]; memory, attention, and decision processes (*n* = 2; 2 low/moderate risk of bias) [68, 92]; goals (*n* = 1; 1 low risk of bias) [84]; and intentions (*n* = 1; 1 moderate risk of bias) [78].

Facilitators that promoted adhering to varenicline include: social influences (*n* = 4; 4 low/moderate risk of bias) [37, 41, 70, 79]; knowledge (*n* = 3; 2 low/moderate risk of bias) [37, 41, 58]; beliefs about capabilities (*n* = 3; 2 moderate risk of bias) [35, 58, 66]; environmental context and resources (*n* = 3; 3 low/moderate risk of bias) [79, 84, 85]; and behavioural regulation (*n* = 1; 1 low risk of bias) [93].

**Fig. 2:**
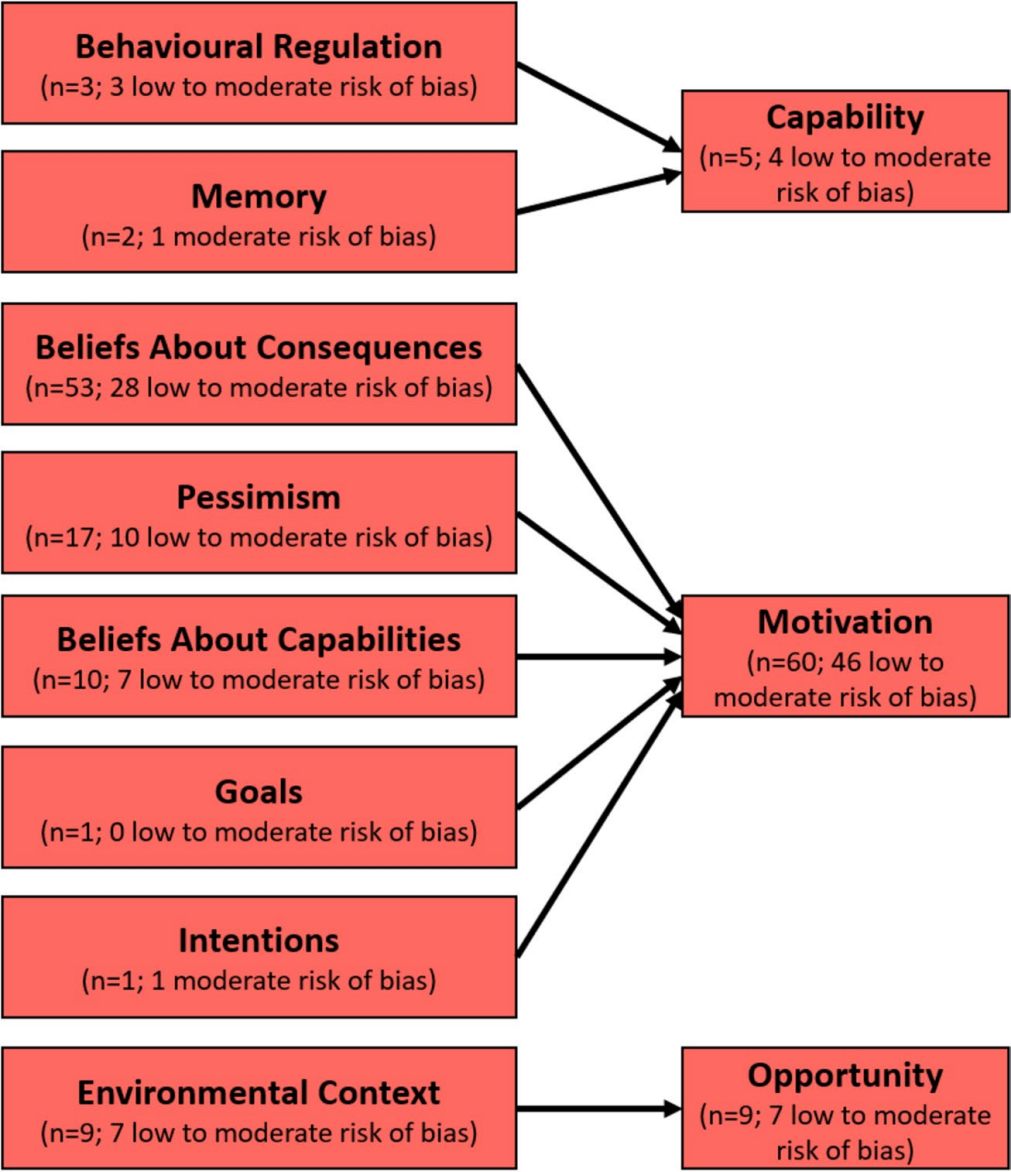
Barriers to varenicline adherence

**Fig. 3:**
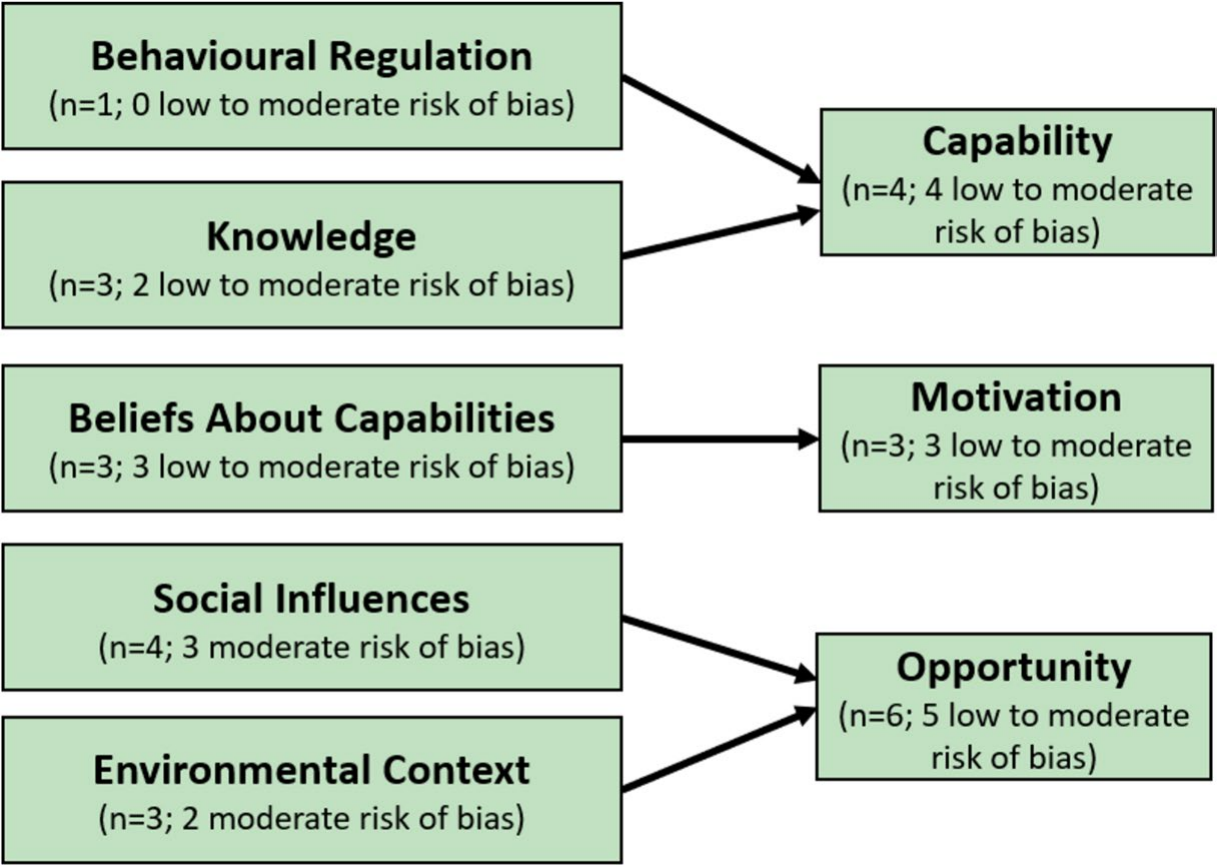
Facilitators to varenicline adherence

There were few instances where modifiable determinants related to ‘goals’ and ‘intentions’ were reported. When they were reported it was always as barriers. On the other hand, “knowledge and “social influence” were only mentioned as facilitators (Figs. 2 and 3). Table 2 provides a summary of the barriers and facilitators to varenicline adherence according to the Theoretical Domains Framework.

## Behaviour change techniques

Only nine studies included in this review reported on behaviour change techniques associated with varenicline adherence [37, 41, 43, 56, 58, 66, 89, 92, 93]. Among these studies, two studies reported statistical significance in regards to improving varenicline adherence [43, 58], two studies were not statistically significant [37, 66], and five studies did not report statistical significance [41, 56, 66, 89, 93].

The most common behaviour change techniques (BCTs) implemented in the studies for improving varenicline adherence were social support (*n* = 6) [37, 41, 43, 56, 66, 93]; feedback and monitoring (*n* = 5) [41, 43, 56, 66, 93]; and shaping knowledge (*n* = 4) [37, 41, 58, 89]. Other BCTs that were mentioned included goals and planning (*n* = 2) [56, 89]; regulation (*n* = 2) [56, 89]; and self-belief (*n* = 1) [58]. Given that so few studies reported statistical significance, we did not identify any trends indicating which BCTs were promising. Table 3 provides a summary of the BCTs used in these studies.

**Table 3:** Behaviour change techniques used to improve varenicline adherence

Behaviour Change Technique	Effectiveness at Improving Varenicline Adherence	Study	Summary of Theme
Goals and planning	Not reported (*n* = 2)	(Purvis et al., 2009) [89]; (Rohsenow et al., 2017) [56]	*Not reported* **1.3 Goal setting (outcome)** Participants received counselling on planning a quit date, the study medication, dosages, and adherence [88] **1.2 Problem solving** Mini counselling sessions were given multiple times to inform participants about a self-help handbook, cognitive behavioural techniques, possible medication issues, and problem-solving methods around barriers [55]
Feedback and monitoring	Effective (*n* = 1) Ineffective (*n* = 1) Not reported (*n* = 3)	(Halperin A.C. et al., 2009) [43]; (Gong J. et al., 2016) [41]; Gordon J.S. et al., 2017) [93]; (Tseng et al., 2017) [66]; (Rohsenow et al., 2017) [56]	*Effective* **2.2 Feedback on behaviour** Participants who had access to individualized counselling and had a counsellor proactively call them to check in and ask questions had better varenicline adherence compared to participants who only had access to web-based forums to ask questions and had to call counsellors themselves for inquiries about symptom management [42] *Ineffective* **2.2 Feedback on behaviour** Combining aspects of both motivational interviewing and cognitive behavioural therapy through phone sessions where a counsellor informed the participant about withdrawal, coping strategies, possible triggers, and the overall study drug. This counselling did not increase varenicline adherence since most of the sessions did not specifically focus on encouraging adherence [65] *Not reported* **2.2 Feedback on behaviour** Using an app that encourages participants to adhere to their medication by giving individualized feedback and tracking their medication usage and information [92]. Mini counselling sessions were given multiple times to inform participants about a self-help handbook, cognitive behavioural techniques, possible medication issues, and problem-solving methods around barriers [55] **2.7 Feedback on outcome(s) of behaviour** Addressing any possible relapse episodes [40]
Social support	Effective (*n* = 1) Mixed results (participants increased their knowledge, skills, or motivation but there is no sign that it improved medication adherence; *n* = 1) Not reported (*n* = 4)	(Halperin A.C. et al., 2009) [43]; (Gong J. et al., 2016) [41]; Gordon J.S. et al., 2017) [93]; Tseng et al., 2017) [66]; (Rohsenow et al., 2017) [56]; (Ebbert et al., 2021) [37]	*Effective* **3.1 Social support (unspecified)** Participants who had access to individualized counselling and had a counselor proactively call them to check in and ask questions had better varenicline adherence compared to participants who only had access to web-based forums to ask questions and had to call counsellors themselves for inquiries about symptom management [42] *Mixed results* **3.1 Social support (unspecified)** Participants who had frequent behavioural counselling that included information and education about the smoking cessation drug had lower rates of medication discontinuation even when experiencing adverse effects, compared to those without counselling [36] *Not reported* **3.1 Social support (unspecified)** Using an app that encourages participants to adhere to their medication by giving individualized feedback and tracking their medication usage and information [92]. Using text message reminders for taking medication and providing motivational and supportive messages. As well as combining aspects of both motivational interviewing and cognitive behavioural therapy through phone sessions where a counsellor informed the participant about withdrawal, coping strategies, possible triggers, and the overall study drug. This type of counselling did not increase varenicline adherence since most of the sessions did not specifically focus on encouraging medication adherence. For text messaging, it may be more useful to have bidirectional messaging between the counsellor and the participant to increase varenicline adherence [65] **3.3 Social support (emotional)** Calling participants to encourage and motivate them to continue with the cessation program through individualized counselling [40]. Mini counselling sessions were given multiple times to inform participants about a self-help handbook, cognitive behavioural techniques, possible medication issues, and problem-solving methods around barriers [55]
Shaping knowledge	Effective (*n* = 1) Mixed results (participants increased their knowledge, skills, or motivation but there is no sign that it improved medication adherence; *n* = 1) Not reported (*n* = 2)	(Gong J. et al., 2016) [41]; Purvis et al., 2009) [89]; (Shelley et al., 2015) [58]; (Ebbert et al., 2021) [37]	*Effective* **4.1 Instruction on how to perform the behavior** Receiving information on varenicline had a positive effect on medication adherence [57] *Mixed results* **4.3 Re-attribution** Participants who had frequent behavioural counselling that included information and education about the smoking cessation drug had lower rates of medication discontinuation even when experiencing adverse effects, compared to those without counselling [36] *Not reported* **4.1 Instruction on how to perform a behaviour; 5.1 Information about health consequences** Counselling sessions discussed the benefits of medication adherence and smoking cessation, potential side effects of the medication, along with the consequences of smoking [40] **4.3 Re-attribution** Participants were given counselling on planning a quit date, the study medication, dosages, and adherence [88]
Regulation	Not reported (*n* = 2)	(Purvis et al., 2009) [89]; (Rohsenow et al., 2017) [56]	*Not reported* **11.1 Pharmacological support** Participants were given counselling on planning a quit date, the study medication, dosages, and adherence [88]. Mini counselling sessions were given multiple times to inform participants about a self-help handbook, cognitive behavioural techniques, possible medication issues, and problem-solving methods around barriers [55]
Self-belief	Effective (*n* = 1)	(Shelley et al., 2015) [58]	*Effective* **15.3 Focus on past success** Varenicline self-efficacy, specific attitudes/beliefs, and information on varenicline had a positive effect on adherence [57]

## Discussion

The goals for this rapid review were to identify: (1) the facilitators and barriers to adhering to varenicline; and (2) the active ingredients utilized in the intervention for varenicline adherence. The results of the review will be used by the authors to help design a healthbot aimed at helping people adhere to their varenicline regimen.

The current review identified 61 studies that identified barriers and/or facilitators to varenicline adherence. Of the 61 studies, nine explicitly mentioned behaviour change techniques used to help with varenicline adherence. Similar to what other evidence syntheses have found on medication adherence [95], our review found a greater emphasis on the barriers than on the facilitators.

By using the TDF framework to extract and analyze the data, we saw that there are eight domains that act as barriers to varenicline adherence (behavioural regulation, memory, goals, intentions, beliefs about capabilities, beliefs about consequences, optimism/pessimism, and environmental context) and five domains that act as facilitators (knowledge, behavioural regulation, beliefs about capabilities, social influences, and environmental context). In this review, side effects were coded under the domains of ‘beliefs about consequences’ since we assumed it is not the side effects per se that influence medication adherence but more of an individual’s acceptance that the medication may cause some unpleasant side effects (such as nausea, sleep disturbances). Under this assumption, the patient’s initial belief that varenicline will lead to side effects may discourage them from beginning their varenicline treatment.

Our results align with other studies, which identified side effects, especially nausea, as a major determinant to varenicline adherence [34, 52]. Additionally, our results are in line with a review investigating factors influencing adherence to Nicotine Replacement Therapy among individuals aiming to quit smoking [96]. This review also identified beliefs about consequences, behavioural regulation, memory, intentions, beliefs about capabilities and environmental context as a significant determinant to medication adherence [96]. Researchers studying different populations (i.e. people with bio-polar disorder, diabetes), have also found beliefs about consequences as a significant determinant to medication adherence [95, 97].

With the exception of the role of optimism/pessimism as a determinant, our findings are similar to other studies using the TDF to understand the determinant of medication adherence [95, 98–100]. In our review, we identified 17 studies reporting pessimism as a barrier to varenicline adherence, which contrasts with the results of other reviews which did not identify pessimism/optimism as a determinant to medication adherence [95, 101]. While it might be a unique case that pessimism is a determinant for varenicline adherence and not to other medications, it is more likely that the difference is due to decisions on how to code certain determinants. Several researchers (who did not use the TDF in their studies) have identified pessimism as an important domain for medication adherence [102–104].

Similar to what other researchers have shown, we found that providing social support, feedback and monitoring, and shaping knowledge were the most common BCTs used to help people adhere to their medication regimen [105–107].

### Strengths and limitations

Our search strategy was comprehensive and was developed with the help of an experienced health sciences research librarian. The included studies were conducted in several countries, and there was representation from all continents, with the exception of Antarctica. In addition, the included studies used a variety of study designs, allowing for a comprehensive list of modifiable determinants of varenicline adherence to be identified. However, due to the nature of rapid reviews, some relevant studies may not have been captured (e.g., exclusion of non-English publications, proceedings and relevant information in the gray literature).

Utilizing the TDF to organize our data, we were able to focus on modifiable determinants and, at the same time, map them to a well-defined theory of behaviour change. However, as mentioned earlier, there were some levels of subjectivity in coding for a few determinants.

Given that very few studies reported BCTs, and of those that did, most did not report the statistical significance of their results, we were unable to examine trends on what BCTs are promising when targeting varenicline adherence.

## Conclusion

Using the TDF framework, our analysis revealed eight domains as barriers (behavioral regulation, memory, goals, intentions, beliefs about capabilities, beliefs about consequences, optimism/pessimism, and environmental context) and five domains as facilitators (knowledge, behavioral regulation, beliefs about capabilities, social influences, and environmental context) to varenicline adherence. The insights into these barriers and facilitators provide valuable guidance for healthcare providers and decision-makers in shaping the design and delivery of smoking cessation services incorporating varenicline. Future work will explore how a healthbot [108, 109] could address the barriers identified in this review.

### Supplementary Information

**Supplementary Material 1 **

**Supplementary Material 2 **

**Supplementary Material 3 **

## Data Availability

Any data extracted during the rapid review process can be provided for review. The extracted data analysed during the current study are available from the corresponding author on request.

## Abbreviations

FDA: Food and Drug Administration
NRT: Nicotine Replacement Therapy
MRC: Medical Research Council
BCTTv1: The Behaviour Change Technique Taxonomy version 1
TDF: Theoretical Domains Framework
COM-B: Capability, Opportunity, and Motivation Model of Behaviour
PRISMA: Preferred Reporting Items for Systematic Reviews and Meta-Analyses
CINAHL: Cumulative Index to Nursing and Allied Health Literature
BCTs: Behaviour Change Techniques
RCTs: Randomized controlled trials
MMAT: Mixed Methods Appraisal Tool
CASP: Critical Appraisal Skills Programme
