# Supplementary material for: Identifying determinants of varenicline adherence using the Theoretical Domains framework: a rapid review
Source: BMC Public Health. 2024 Mar 4;24:679. doi: 10.1186/s12889-024-18139-z (PMC10910805; doi:10.1186/s12889-024-18139-z)
Supplement: Supplementary file 2 — Supplementary Material 2 [file 12889_2024_18139_MOESM2_ESM.pdf]

Additional File 2- MEDLINE search strategy

Identifying determinants of varenicline adherence using the Theoretical Domains Framework: a rapid review

Database (searched on May 6, 2022): Ovid MEDLINE: Epub Ahead of Print, In-Process & Other Non-Indexed Citations, Ovid MEDLINE® Daily and Ovid MEDLINE® <1946-Present>

1	Varenicline/	1469
2	Varenicline.ti,ab,kf.	1728
3	(Chantix or Champix).ti,ab,kf.	111
4	or/1-3	1922
5	smoking cessation/ or smoking reduction/ or "tobacco use cessation"/	32619
6	(smoke or smokes or smoking or smoker* or nicotine or tobacco or cigar*).ti,ab,kf,hw.	396484
7	or/5-6	396484
8	treatment adherence and compliance/	922
9	patient acceptance of health care/	53224
10	patient compliance/	60135
11	patient dropouts/	8381
12	patient participation/	28499
13	(retention or attrition or continu* or discontinu* or disengag* or terminat* or dropout* or drop* out* or complet* or finish* or adher* or nonadher* or complian* or noncomplian* or engag* or attend*).ti,kf,hw.	554089
14	((retention or attrition or continu* or discontinu* or disengag* or terminat* or dropout* or drop* out* or complet* or finish* or adher* or nonadher* or complian* or noncomplian* or engag* or attend*) adj3 (treatment* or therap* or program*)).ab.	185862
15	((retention or attrition or continu* or discontinu* or disengag* or terminat* or dropout* or drop* out* or complet* or finish* or adher* or nonadher* or complian* or noncomplian* or engag* or attend*) adj3 (predict* or factor* or characteristic*)).ab.	39046
16	acceptability.ti,ab,kf,hw.	37298
17	(barrier* or facilitator* or mediat* or moderator*).ti,kf,hw.	416189
18	((barrier* or facilitator*) adj3 (treatment* or therap* or retention or attrition or continu* or discontinu* or engag* or disengag* or terminat* or dropout* or drop* out* or complet* or finish* or initiat* or adher* or nonadher* or complian* or noncomplian* or engag* or attend*)).ab.	11978
19	or/8-18	1229780
20	4 and 7 and 19	221
21	limit 20 to (letter or editorial)	2
*22	(Animals/ or Models, Animal/ or Disease Models, Animal/) not Humans/	4966365
*23	((animal or animals or canine* or dog or dogs or feline or hamster* or lamb or lambs or mice or monkey or monkeys or mouse or murine or pig or pigs or piglet* or porcine or primate* or rabbit* or rats or rat or rodent* or sheep* or veterinar*) not (human* or patient*)).ti,kf,jw.	2302274
24	20 not (21 or 22 or 23)	215
25	limit 24 to yr="2006 -Current"	215

*Lines 22-23: Excluding Animal Studies. McGill University Health Centre (MUHC) Libraries, published June 13, 2019. Retrieved from: https://www.muhclibraries.ca/training-and-guides/excluding-animal-studies/
