# Supplementary material for: Identifying determinants of varenicline adherence using the Theoretical Domains framework: a rapid review
Source: BMC Public Health. 2024 Mar 4;24:679. doi: 10.1186/s12889-024-18139-z (PMC10910805; doi:10.1186/s12889-024-18139-z)
Supplement: Supplementary file 3 — Supplementary Material 3 [file 12889_2024_18139_MOESM3_ESM.pdf]

Additional File 3- Data Extraction Template

Data Extraction Template

Question	Options	Notes
**General Information**		
**Extractor's initials**	[text]	e.g. "JD" for Jane Doe
**Study ID**	[text]	
**Last name of first author**	[text]	
**Year of publication**	[text]	e.g. 1980
**Title**	[text]	
**DOI**	[text]	
**Corresponding author contact information**	[text]	e.g. name, affiliation, address, phone number, email address
**1. Was varenicline used for smoking cessation?**	○ Yes<br>○ No	
**2. Are factors (barriers and/or facilitators) associated with only varenicline adherence reported separately?**	○ Yes<br>○ No	
**If you answered "No" to any of the previous two questions, exclude study.**		
**3. a) On what continent did the intervention take place? (If not specified, resort to author affiliations)**	☐ North America<br>☐ Europe<br>☐ Australasia<br>☐ Central America/South America/Caribbean<br>☐ Asia<br>☐ Africa<br>☐ Other: [text]	(Check all that apply)<br><br>North America: includes Canada, USA and Mexico<br><br>Europe: includes Belarus, Latvia, Ukraine, Estonia, Cyprus & west (includes Iceland and Greenland)<br><br>Australasia: limited to Australia, New Guinea, New Zealand, New Caledonia, and neighbouring islands, including the Indonesian islands from Lombok and Sulawesi eastward<br><br>Central America/South America/ Caribbean: includes Caribbean, and all of south and central America<br><br>Asia: Russia, Turkey, middle eastern countries and east
**3. b) If applicable, specify the country, state, or province.**	[text]	Enter "N/A" if not applicable.

**4. What is the study design?**	○ Experimental study: randomized controlled trial<br>○ Experimental study: quasi-experimental<br>○ Observational study: cross sectional<br>○ Observational study: cohort<br>○ Observational study: case control<br>○ Qualitative study<br>○ Other: [text]	Randomized control trial: individuals are allocated at random to a control or intervention group<br><br>Quasi-experimental: used to estimate the causal impact of an intervention on its target population without random assignment<br><br>Cross sectional: measures the prevalence of health outcomes or determinants of health, or both, in a population at a point in time or over a short period<br><br>Cohort: follow a group of exposed and non-exposed individuals to evaluate whether they develop an outcome<br><br>Case control: identified cases are matched with controls and their risk factors are evaluated for an association with outcome<br><br>Qualitative: primarily exploratory research. It is used to gain an understanding of underlying reasons, opinions, and motivations (e.g. focus groups, interviews)
**5. What theories were used to design the intervention?**	☐ N/A<br>☐ Behavioural Therapy<br>☐ The Behaviour Change Approach<br>☐ The Community Organization Approach<br>☐ Social Learning Theory<br>☐ Social Cognitive Theory<br>☐ Self Regulation Model<br>☐ Health Belief Model<br>☐ Social-Ecological Theory<br>☐ Goal Systems Theory<br>☐ Transtheoretical Model<br>☐ Other: [text]	(Check all that apply)<br><br>Behavioural Therapy: structured approach which focuses on measuring what a person is doing and helps them understand how changing their behaviour can lead to positive experiences<br><br>The Behaviour Change Approach: process for planning and implementing a strategic set of interventions and activities to change behaviours<br><br>The Community Organization Approach: method of intervention where a professional change agent helps a community action system composed of individuals, groups, or organizations to engage in planned collective action to deal with a community issue<br><br>Social Learning Theory: proposes that new behaviours can be learned by observing and imitating others

		Social Cognitive Theory: proposes that an individual’s behaviour is guided through observing others within the context of social interactions, experiences, and outside media influences<br><br>Self Regulation Model consists of three constructs: (1) ‘illness perceptions’ (i.e. the beliefs a person holds about their illness), (2) ‘coping responses’/action planning, (3) ‘appraisal’/monitoring of responses. The SRM suggests that individuals search to understand their illness or disease threat by developing an understanding of what the illness is, what it means, its causes, its consequences, how long it will last, and whether it can be cured or controlled<br><br>Health Belief Model consists of four key constructs: ‘perceived barriers’, ‘perceived benefits’, ‘perceived severity’ and ‘perceived susceptibility’. This model suggests that an individual’s thoughts and actions are mainly rational, and the behaviour will be carried out if the perceived threat (severity and susceptibility) is high and perceived benefits outweigh barriers<br><br>Social-Ecological Theory: framework for understanding the multifaceted and interactive effects of personal and environmental factors that determine behaviours<br><br>Goal Systems Theory: offers insight into achievement of goal dynamics through goals (representations of desired end-states) and goal-means (behaviours that can help one accomplish a goal)<br><br>Transtheoretical Model: assesses an individual’s readiness to act on a new behaviour, and provides strategies, or processes of change to guide the individual through the stages of change

<table>
<tr><td>6. a) How was the intervention delivered?</td><td>☐ N/A
☐ Not stated
☐ Face to face
☐ Over the phone
☐ Web
☐ App
☐ SMS text
☐ Mass media
☐ Other: [text]</td><td>Intervention refers to the overall intervention used in the study.

(Check all that apply)</td></tr>
<tr><td>6. b) Briefly describe the intervention</td><td>[text]</td><td>Enter "N/A" if not applicable.</td></tr>
<tr><td>7. What was the end goal of the varenicline treatment?</td><td>○ Quitting
○ Reduction
○ Other: [text]</td><td></td></tr>
<tr><td colspan="3"><h2>Sample Population</h2></td></tr>
<tr><td>8. What is the sample size of participants in the study?</td><td><table><tr><th></th><th>Group</th><th>Sample Size</th></tr><tr><td>Group 1</td><td></td><td></td></tr><tr><td>Group 2</td><td></td><td></td></tr><tr><td>Group 3</td><td></td><td></td></tr><tr><td>Group 4</td><td></td><td></td></tr><tr><td>Group 5</td><td></td><td></td></tr><tr><td>Group 6</td><td></td><td></td></tr><tr><td>Total</td><td></td><td></td></tr></table></td><td>Specify the group in the middle column (e.g., control, intervention)

Enter numerical values in the right column (e.g. 14).

Enter "N/A" if not applicable.</td></tr>
<tr><td>9. Indicate the % female in the study.</td><td><table><tr><th></th><th>Group</th><th>% Female</th></tr><tr><td>Group 1</td><td></td><td></td></tr><tr><td>Group 2</td><td></td><td></td></tr><tr><td>Group 3</td><td></td><td></td></tr><tr><td>Group 4</td><td></td><td></td></tr><tr><td>Group 5</td><td></td><td></td></tr><tr><td>Group 6</td><td></td><td></td></tr><tr><td>Total</td><td></td><td></td></tr></table></td><td>Specify the group in the middle column (e.g. control, intervention).

Enter numerical values in the right column (e.g. 14).

Enter "N/A" if not applicable</td></tr>
<tr><td>10. a) What was the target population of this intervention?</td><td>☐ General public
☐ Pregnant people
☐ Students
☐ Minority groups
☐ Disadvantaged
☐ Cardio patients
☐ Cancer patients
☐ Other: [text]</td><td>(Check all that apply)</td></tr>
<tr><td>10. b) If applicable, specify any relevant details about the target population</td><td>[text]</td><td>Enter "N/A" if not applicable.</td></tr>
<tr><td>11. What tobacco products were the participants using?</td><td>☐ Cigarettes
☐ E-cigarettes
☐ Smokeless tobacco
☐ Other: [text]</td><td>(Check all that apply)</td></tr>
</table>

**12. What age range are the participants of the study?**	☐ Adolescents (<16)<br>☐ Adults (16-64)<br>☐ Elderly (65+)	(Check all that apply)
**13. a) What is the race of the participants in the study?**	☐ White/Caucasian<br>☐ Black or African American<br>☐ Aboriginal or Indigenous<br>☐ Asian/Pacific Islander<br>☐ Hispanic or Latino<br>☐ Not stated<br>☐ Other: [text]	(Specify for both intervention and control in the following question)<br><br>(Check all that apply)
**13. b) If applicable, specify any relevant details about the race of the participants.**	[text]	Enter "N/A" if not applicable.
**14. a) Was any additional demographic information about the study participants captured?**	☐ Socioeconomic status<br>☐ Occupation<br>☐ Education<br>☐ Religion/culture<br>☐ Not stated<br>☐ Other: [text]	(Check all that apply)
**14. b) If applicable, specify the additional demographic information for both intervention and control.**	[text]	Enter "N/A" if not applicable.
**Barriers Identified**		
**15. What types of barriers are described in the study?**	☐ N/A<br>☐ (B.i) Adverse effects<br>☐ (B.1) Knowledge<br>☐ (B.2) Skills<br>☐ (B.3) Social/professional role and identity<br>☐ (B.4) Beliefs about capabilities<br>☐ (B.5) Optimism<br>☐ (B.6) Beliefs about Consequences<br>☐ (B.7) Reinforcement<br>☐ (B.8) Intentions<br>☐ (B.9) Goals<br>☐ (B.10) Memory, attention and decision processes<br>☐ (B.11) Environmental context and resources<br>☐ (B.12) Social influences<br>☐ (B.13) Emotion<br>☐ (B.14) Behavioural regulation	(Check all that apply)<br><br>(B.1) Knowledge: An awareness of the existence of something<br><br>(B.2) Skills: An ability or proficiency acquired through practice<br><br>(B.3) Social/professional role and identity: A coherent set of behaviours and displayed personal qualities of an individual in a social or work setting<br><br>(B.4) Beliefs about capabilities: Acceptance of the truth, reality or validity about an ability, talent or facility that a person can put to constructive use<br><br>(B.5) Optimism: The confidence that things will happen for the best or that desired goals will be attained<br><br>(B.6) Beliefs about Consequences: Acceptance of the truth, reality, or validity about outcomes of a behaviour in a given situation

		(B.7) Reinforcement: Increasing the probability of a response by arranging a dependent relationship, or contingency, between the response and a given stimulus<br><br>(B.8) Intentions: A conscious decision to perform a behaviour or a resolve to act in a certain way<br><br>(B.9) Goals: Mental representations of outcomes or end states that an individual wants to achieve<br><br>(B.10) Memory, attention and decision processes: The ability to retain information, focus selectively on aspects of the environment and choose between two or more alternatives<br><br>(B.11) Environmental context and resources: Any circumstance of a person's situation or environment that discourages or encourages the development of skills and abilities, independence, social competence and adaptive behaviour<br><br>(B.12) Social influences: Those interpersonal processes that can cause individuals to change their thoughts, feelings, or behaviours<br><br>(B.13) Emotion: A complex reaction pattern, involving experiential, behavioural, and physiological elements, by which the individual attempts to deal with a personally significant matter or event<br><br>(B.14) Behavioural regulation: Anything aimed at managing or changing objectively observed or measured actions
**If applicable, describe the barrier(s). If not applicable, move to "Facilitators Identified" questions.**		
**(B.i) Adverse effects**	☐ Nausea<br>☐ Constipation<br>☐ Flatulence<br>☐ Insomnia<br>☐ Abnormal dreams<br>☐ Fatigue<br>☐ Headache<br>☐ Vomiting<br>☐ Other: [text]	

**(B.1) Knowledge**	[text]	(An awareness of the existence of something)<br><br>Knowledge (including knowledge of condition/scientific rationale)<br>Procedural knowledge<br>Knowledge of task environment
**(B.2) Skills**	[text]	(An ability or proficiency acquired through practice)<br><br>Skills<br>Skills development<br>Competence<br>Ability<br>Interpersonal skills<br>Practice<br>Skill assessment
**(B.3) Social/professional role and identity**	[text]	(A coherent set of behaviours and displayed personal qualities of an individual in a social or work setting)<br><br>Professional identity<br>Professional role<br>Social identity<br>Identity<br>Professional boundaries<br>Professional confidence<br>Group identity<br>Leadership<br>Organisational commitment
**(B.4) Beliefs about capabilities**	[text]	(Acceptance of the truth, reality or validity about an ability, talent or facility that a person can put to constructive use)<br><br>Self-confidence<br>Perceived competence<br>Self-efficacy<br>Perceived behavioural control<br>Beliefs<br>Self-esteem<br>Empowerment<br>Professional confidence
**(B.5) Optimism**	[text]	(The confidence that things will happen for the best or that desired goals will be attained)<br><br>Optimism<br>Pessimism<br>Unrealistic optimism<br>Identity

**(B.6) Beliefs about Consequences**	[text]	(Acceptance of the truth, reality, or validity about outcomes of a behaviour in a given situation)<br><br>Beliefs<br>Outcome expectancies<br>Characteristics of outcome expectancies<br>Anticipated regret<br>Consequents
**(B.7) Reinforcement**	[text]	(Increasing the probability of a response by arranging a dependent relationship, or contingency, between the response and a given stimulus)<br><br>Rewards (proximal/distal, valued/not valued, probable/improbable)<br>Incentives<br>Punishment<br>Consequents<br>Reinforcement<br>Contingencies<br>Sanctions
**(B.8) Intentions**	[text]	(A conscious decision to perform a behaviour or a resolve to act in a certain way)<br><br>Stability of intentions<br>Stages of change model<br>Transtheoretical model and stages of change
**(B.9) Goals**	[text]	(Mental representations of outcomes or end states that an individual wants to achieve)<br><br>Goals (distal/proximal)<br>Goal priority<br>Goal/target setting<br>Goals (autonomous/controlled)<br>Action planning<br>Implementation intention
**(B.10) Memory, attention and decision processes**	[text]	(The ability to retain information, focus selectively on aspects of the environment and choose between two or more alternatives)<br><br>Memory<br>Attention<br>Attention control<br>Decision making<br>Cognitive overload/tiredness

**(B.11) Environmental context and resources**	[text]	(Any circumstance of a person's situation or environment that discourages or encourages the development of skills and abilities, independence, social competence and adaptive behaviour)<br><br>Environmental stressors<br>Resources/material resources<br>Organisational culture/climate<br>Salient events/critical incidents<br>Person × environment interaction<br>Barriers and facilitators
**(B.12) Social influences**	[text]	(Those interpersonal processes that can cause individuals to change their thoughts, feelings, or behaviours)<br><br>Social pressure<br>Social norms<br>Group conformity<br>Social comparisons<br>Group norms<br>Social support<br>Power<br>Intergroup conflict<br>Alienation<br>Group identity<br>Modelling
**(B.13) Emotion**	[text]	(A complex reaction pattern, involving experiential, behavioural, and physiological elements, by which the individual attempts to deal with a personally significant matter or event)<br><br>Fear<br>Anxiety<br>Affect<br>Stress<br>Depression<br>Positive/negative affect<br>Burn-out
**(B.14) Behavioural regulation**	[text]	(Anything aimed at managing or changing objectively observed or measured actions)<br><br>Self-monitoring<br>Breaking habit<br>Action planning

Facilitators Identified		
**16. What types of facilitators are described in the study?**	☐ N/A<br>☐ (F.1) Knowledge<br>☐ (F.2) Skills<br>☐ (F.3) Social/professional role and identity<br>☐ (F.4) Beliefs about capabilities<br>☐ (F.5) Optimism<br>☐ (F.6) Beliefs about Consequences<br>☐ (F.7) Reinforcement<br>☐ (F.8) Intentions<br>☐ (F.9) Goals<br>☐ (F.10) Memory, attention and decision processes<br>☐ (F.11) Environmental context and resources<br>☐ (F.12) Social influences<br>☐ (F.13) Emotion<br>☐ (F.14) Behavioural regulation	(Check all that apply)<br><br>(F.1) Knowledge: An awareness of the existence of something<br><br>(F.2) Skills: An ability or proficiency acquired through practice<br><br>(F.3) Social/professional role and identity: A coherent set of behaviours and displayed personal qualities of an individual in a social or work setting<br><br>(F.4) Beliefs about capabilities: Acceptance of the truth, reality or validity about an ability, talent or facility that a person can put to constructive use<br><br>(F.5) Optimism: The confidence that things will happen for the best or that desired goals will be attained<br><br>(F.6) Beliefs about Consequences: Acceptance of the truth, reality, or validity about outcomes of a behaviour in a given situation<br><br>(F.7) Reinforcement: Increasing the probability of a response by arranging a dependent relationship, or contingency, between the response and a given stimulus<br><br>(F.8) Intentions: A conscious decision to perform a behaviour or a resolve to act in a certain way<br><br>(F.9) Goals: Mental representations of outcomes or end states that an individual wants to achieve<br><br>(F.10) Memory, attention and decision processes: The ability to retain information, focus selectively on aspects of the environment and choose between two or more alternatives<br><br>(F.11) Environmental context and resources: Any circumstance of a person's situation or environment that discourages or encourages the development of skills and abilities, independence, social competence and adaptive behaviour

		(F.12) Social influences: Those interpersonal processes that can cause individuals to change their thoughts, feelings, or behaviours<br><br>(F.13) Emotion: A complex reaction pattern, involving experiential, behavioural, and physiological elements, by which the individual attempts to deal with a personally significant matter or event<br><br>(F.14) Behavioural regulation: Anything aimed at managing or changing objectively observed or measured actions
**If applicable, describe the facilitator(s). If not applicable, move to "Activities (BCTs)" questions.**		
**(F.1) Knowledge**	[text]	(An awareness of the existence of something)<br><br>Knowledge (including knowledge of condition/scientific rationale)<br>Procedural knowledge<br>Knowledge of task environment
**(F.2) Skills**	[text]	(An ability or proficiency acquired through practice)<br><br>Skills<br>Skills development<br>Competence<br>Ability<br>Interpersonal skills<br>Practice<br>Skill assessment
**(F.3) Social/professional role and identity**	[text]	(A coherent set of behaviours and displayed personal qualities of an individual in a social or work setting)<br><br>Professional identity<br>Professional role<br>Social identity<br>Identity<br>Professional boundaries<br>Professional confidence<br>Group identity<br>Leadership<br>Organisational commitment

**(F.4) Beliefs about capabilities**	[text]	(Acceptance of the truth, reality or validity about an ability, talent or facility that a person can put to constructive use)<br><br>Self-confidence<br>Perceived competence<br>Self-efficacy<br>Perceived behavioural control<br>Beliefs<br>Self-esteem<br>Empowerment<br>Professional confidence
**(F.5) Optimism**	[text]	(The confidence that things will happen for the best or that desired goals will be attained)<br><br>Optimism<br>Pessimism<br>Unrealistic optimism<br>Identity
**(F.6) Beliefs about Consequences**	[text]	(Acceptance of the truth, reality, or validity about outcomes of a behaviour in a given situation)<br><br>Beliefs<br>Outcome expectancies<br>Characteristics of outcome expectancies<br>Anticipated regret<br>Consequents
**(F.7) Reinforcement**	[text]	(Increasing the probability of a response by arranging a dependent relationship, or contingency, between the response and a given stimulus)<br><br>Rewards (proximal/distal, valued/not valued, probable/improbable)<br>Incentives<br>Punishment<br>Consequents<br>Reinforcement<br>Contingencies<br>Sanctions
**(F.8) Intentions**	[text]	(A conscious decision to perform a behaviour or a resolve to act in a certain way)<br><br>Stability of intentions<br>Stages of change model<br>Transtheoretical model and stages of change

**(F.9) Goals**	[text]	(Mental representations of outcomes or end states that an individual wants to achieve)<br><br>Goals (distal/proximal)<br>Goal priority<br>Goal/target setting<br>Goals (autonomous/controlled)<br>Action planning<br>Implementation intention
**(F.10) Memory, attention and decision processes**	[text]	(The ability to retain information, focus selectively on aspects of the environment and choose between two or more alternatives)<br><br>Memory<br>Attention<br>Attention control<br>Decision making<br>Cognitive overload/tiredness
**(F.11) Environmental context and resources**	[text]	(Any circumstance of a person's situation or environment that discourages or encourages the development of skills and abilities, independence, social competence and adaptive behaviour)<br><br>Environmental stressors<br>Resources/material resources<br>Organisational culture/climate<br>Salient events/critical incidents<br>Person × environment interaction<br>Barriers and facilitators
**(F.12) Social influences**	[text]	(Those interpersonal processes that can cause individuals to change their thoughts, feelings, or behaviours)<br><br>Social pressure<br>Social norms<br>Group conformity<br>Social comparisons<br>Group norms<br>Social support<br>Power<br>Intergroup conflict<br>Alienation<br>Group identity<br>Modelling

**(F.13) Emotion**	[text]	(A complex reaction pattern, involving experiential, behavioural, and physiological elements, by which the individual attempts to deal with a personally significant matter or event)<br><br>Fear<br>Anxiety<br>Affect<br>Stress<br>Depression<br>Positive/negative affect<br>Burn-out
**(F.14) Behavioural regulation**	[text]	(Anything aimed at managing or changing objectively observed or measured actions)<br><br>Self-monitoring<br>Breaking habit<br>Action planning
**Activities (BCTs)**		
**17. What types of activities did the intervention use?**	☐ N/A<br>☐ (A.1) Goals and planning<br>☐ (A.2) Feedback and monitoring<br>☐ (A.3) Social support<br>☐ (A.4) Shaping knowledge<br>☐ (A.5) Natural consequences<br>☐ (A.6) Comparison of behaviour<br>☐ (A.7) Associations<br>☐ (A.8) Repetition and substitution<br>☐ (A.9) Comparison of outcomes<br>☐ (A.10) Reward and threat<br>☐ (A.11) Regulation<br>☐ (A.12) Antecedents<br>☐ (A.13) Identity<br>☐ (A.14) Scheduled consequences<br>☐ (A.15) Self-belief<br>☐ (A.16) Covert learning	Intervention refers to any treatment with the aim of helping people adhere to varenicline.<br><br>(Refer to handout for the definitions of BCTs. Check all that apply)
**If applicable, describe the activities the intervention used. If not applicable, move to "Outcomes" questions.**		

**(A.1) Goals and planning**	[text]	1.1 Goal setting (behavior)<br>1.2 Problem solving<br>1.3 Goal setting (outcome)<br>1.4 Action planning<br>1.5 Review behavior goal(s)<br>1.6 Discrepancy between current behavior and goal<br>1.7 Review outcome goal(s)<br>1.8 Behavioral contract<br>1.9 Commitment
**(A.2) Feedback and monitoring**	[text]	2.1 Monitoring of behavior by others without feedback<br>2.2 Feedback on behavior<br>2.3 Self-monitoring of behavior<br>2.4 Self-monitoring of outcome(s) of behavior<br>2.5 Monitoring outcome(s) of behavior by others without feedback<br>2.6 Biofeedback<br>2.7 Feedback on outcome(s) of behavior
**(A.3) Social support**	[text]	3.1 Social support (unspecified)<br>3.2 Social support (practical)<br>3.3 Social support (emotional)
**(A.4) Shaping knowledge**	[text]	4.1 Instruction on how to perform a behavior<br>4.2 Information about antecedents<br>4.3 Re-attribution<br>4.4 Behavioral experiments
**(A.5) Natural consequences**	[text]	5.1 Information about health consequences<br>5.2 Salience of consequences<br>5.3 Information about social and environmental consequences<br>5.4 Monitoring of emotional consequences<br>5.5 Anticipated regret<br>5.6 Information about emotional consequences
**(A.6) Comparison of behaviour**	[text]	6.1 Demonstration of the behavior<br>6.2 Social comparison<br>6.3 Information about others' approval
**(A.7) Associations**	[text]	7.1 Prompts/cues<br>7.2 Cue signalling reward<br>7.3 Reduce prompts/cues<br>7.4 Remove access to the reward<br>7.5 Remove aversive stimulus<br>7.6 Satiation<br>7.7 Exposure<br>7.8 Associative learning

**(A.8) Repetition and substitution**	[text]	8.1 Behavioral practice/ rehearsal<br>8.2 Behavior substitution<br>8.3 Habit formation<br>8.4 Habit reversal<br>8.5 Overcorrection<br>8.6 Generalisation of a target behavior<br>8.7 Graded tasks
**(A.9) Comparison of outcomes**	[text]	9.1 Credible source<br>9.2 Pros and cons<br>9.3 Comparative imagining of future outcomes
**(A.10) Reward and threat**	[text]	10.1 Material incentive (behavior)<br>10.2 Material reward (behavior)<br>10.3 Non-specific reward<br>10.4 Social reward<br>10.5 Social incentive<br>10.6 Non-specific incentive<br>10.7 Self-incentive<br>10.8 Incentive (outcome)<br>10.9 Self-reward<br>10.10 Reward (outcome)<br>10.11 Future punishment
**(A.11) Regulation**	[text]	11.1 Pharmacological support<br>11.2 Reduce negative emotions<br>11.3 Conserving mental resources<br>11.4 Paradoxical instructions
**(A.12) Antecedents**	[text]	12.1 Restructuring the physical environment<br>12.2 Restructuring the social environment<br>12.3 Avoidance/reducing exposure to cues for the behavior<br>12.4 Distraction<br>12.5 Adding objects to the environment<br>12.6 Body changes
**(A.13) Identity**	[text]	13.1 Identification of self as role model<br>13.2 Framing/reframing<br>13.3 Incompatible beliefs<br>13.4 Valued self-identity<br>13.5 Identity associated with changed behavior

**(A.14) Scheduled consequences**	[text]	14.1 Behavior cost<br>14.2 Punishment<br>14.3 Remove reward<br>14.4 Reward approximation<br>14.5 Rewarding completion<br>14.6 Situation-specific reward<br>14.7 Reward incompatible behavior<br>14.8 Reward alternative behavior<br>14.9 Reduce reward frequency<br>14.10 Remove punishment
**(A.15) Self-belief**	[text]	15.1 Verbal persuasion about capability<br>15.2 Mental rehearsal of successful performance<br>15.3 Focus on past success<br>15.4 Self-talk
**(A.16) Covert learning**	[text]	16.1 Imaginary punishment<br>16.2 Imaginary reward<br>16.3 Vicarious consequences
**Outcomes**		
**18. Definition of varenicline adherence**	[text]	Enter "N/A" if not applicable.
**19. Adherence outcome measures**	☐ Self report<br>☐ Pill count<br>☐ Medication possession ratios (MPR)<br>☐ Proportion of days covered (PDC)<br>☐ Electronic monitoring using medication event monitoring systems (MMSE)<br>☐ Other: [text]	
**20. Degree of non-adherence**	[text]	Briefly describe the degree of non-adherence (e.g., discontinuation)
**21. Additional comments**	[text]	
